# Soil properties and plant species can predict population size and potential introduction sites of the endangered orchid *Cypripedium calceolus*

**DOI:** 10.1007/s11104-023-05945-4

**Published:** 2023-02-16

**Authors:** Olivia Rusconi, Théo Steiner, Claire Le Bayon, Sergio Rasmann

**Affiliations:** Institute of Biology, University of Neuchâtel, Rue Emile-Argand 11, 2000 Neuchâtel, Switzerland

**Keywords:** Edaphic properties, Indicator species, Orchidaceae, Plant conservation biology, Red-list species, Vegetation alliances

## Abstract

**Background and Aims:**

To counteract the ongoing worldwide biodiversity loss, conservation actions are required to re-establish populations of threatened species. Two key factors predominantly involved in finding the most suitable habitats for endangered plant species are the surrounding plant community composition and the physicochemical parameters of the soil rooting zone. However, such factors are likely to be context- and species-dependent, so it remains unclear to what extent they influence the performance of target species.

**Methods:**

We studied large and small Swiss populations of the endangered orchid *Cypripedium calceolus*. We measured functional traits related to *C. calceolus* plant and population performance (clonal patch area, plant height, number, of leaf, stems, flowers and fruits), realized vegetation surveys, soil profile analyses, and tested for relationships between plant traits and the surrounding vegetation structure or soil physicochemical parameters.

**Results:**

Large populations contained bigger patches with more stems and leaves, and produced more flower per individual than small populations. Neither vegetation alliances nor soil classes per se could predict *C. calceolus* functional traits and population size. However, functional traits explaining population performance and size were related to specific soil parameters (soil organic matter content, pH and phosphorus), in addition to a combination of presence-absence of plant indicator species, relating to ecotones between forests and clearings.

**Conclusion:**

We show that even for species that can grow across a wide range of vegetation groups both indicator species and specific soil parameters can be used to assess the most favourable sites to implement (re)-introduction actions.

**Supplementary Information:**

The online version contains supplementary material available at 10.1007/s11104-023-05945-4.

## Introduction

One-fifth of plant species worldwide are estimated to be at risk of extinction (Willis 2017) due to a number of factors, notably human-driven habitat perturbation and climate change (Barnosky et al. 2011; Ceballos et al. 2017). Such biodiversity erosion has led to an urgent need for conservation actions, particularly via habitat protection (Barnosky et al. 2017). Despite this, habitat loss, fragmentation and change mean that the survival and persistence of rare plant populations can seldom be achieved by natural recruitment and dispersal alone. Instead, species conservation must rely on active reintroduction efforts in previously selected and appropriate habitat types (Seddon 2010). Consequently, to develop efficient conservation strategies for plants, a necessary prerequisite is to gather the best knowledge of the most important ecological factors that might influence the survival and the vitality of a target species (Godefroid et al. 2011; Heywood and Iriondo 2003; Seddon et al. 2007). The field of conservation science thus aims to act rapidly to understand the causes of each species disappearance and find suitable habitats for implementing successful (re)-introduction plans (Pimm et al. 2014; Primack 2014; Swarts and Dixon 2009).

For plant establishment and perpetuation, soil (edaphic) properties and the composition of surrounding vegetation communities are critical parameters that need to be evaluated (Antonovics and Bradshaw 1970; Rusconi et al. 2022). Accordingly, it is crucial to identify the potential links between soil properties, vegetation communities and the success of the focal endangered plant species (Godefroid et al. 2011). For instance, Vittoz et al. (2006), by analysing the edaphic conditions, hydrology, microtopography, and vegetation types of the niche of the endangered herbaceous plant *Saxifraga hirculus*, identified four major themes of conservation activities, namely grazing, mowing, reintroduction and substrate management. Similarly, through field surveys and herbarium analyses, Ren et al. (2010) were able to decipher the ecological requirements of the endangered *Primulina tabacum* in China and to identify suitable reintroduction sites. Moreover, factors determining the survival of the endangered endemic limestone-associated *Purshia subintegra* were uncovered by measuring 16 environmental factors, including soil and vegetation variables, in natural and experimental reintroduction sites (Maschinski et al. 2004). Therefore, the best chance of finding suitable habitats for endangered species (re)-introductions plans is to take a holistic approach combining measures of two of the predominant facets of terrestrial ecosystems: soil and vegetation properties.

The orchids (Orchidaceae) are among the most threatened plant families. Orchids have been subjected to intense collection because of their intrinsic beauty, while their complex and delicate life cycle has led to slow the recovery, or even disappearance, of populations (Hinsley et al. 2017; Thomas 2006). A well-known emblematic and patrimonial orchid growing throughout Eurasia is the Lady’s slipper orchid *Cypripedium calceolus* L. (Fay and Taylor 2015). Although *C. calceolus* has a wide Eurasian distribution, ranging from the United Kingdom to the Pacific Ocean (Kull 1999), populations are in fact very scattered throughout its range (Devillers-Terschuren 1999). More so, the number and size of *C. calceolus* populations have drastically declined over the last decades in several European countries (Devillers-Terschuren 1999; Kull 1999). *C. calceolus* has been attributed to different conservation statuses across its distribution, being for example of Least Concern (LC) at a global scale, but marked as Vulnerable (VU) in Switzerland and Critically Endangered (CR) in the UK (Bornand et al. 2016; Gargiulo et al. 2018; Bilz 2011; Stroh et al. 2014).

The complexity and the eco-physiological particularities of this orchid, in addition to its patrimonial significance (Devillers-Terschuren 1999), have contributed to this species general decline (Gargiulo et al. 2021; Swarts and Dixon 2009). The physiological reasons for this decline might be that *C. calceolus* development is slow, and its life expectancy is long, theoretically up to more than 30 years (Käsermann and Moser 1999). From an ecological point of view, *C. calceolus* is endangered because its ecological requirements are mainly supposed to depend on an integrated sum of multiple parameters (Swarts and Dixon 2017). It is thought that *C. calceolus* requires a combination of three key ecological factors to thrive: (i) light intensity, (ii) soil moisture and (iii) soil base richness (Devillers-Terschuren 1999). For light intensity, *C. calceolus* populations should be primarily found in shady, deciduous, and mixed woodland forest types with a high canopy and very few bushes. This allows solar radiation to reach this species indirectly (Kull 1999; Rusconi et al. 2022). However, *C. calceolus* populations have also been shown to thrive in full sunlight at high elevations (Devillers-Terschuren 1999; Kull 1999). In Switzerland, *C. calceolus* has been found growing in as many as up to 14 different vegetation alliances, with a preference for the *Cephalanthero-Fagenion* (xerothermophilous beech forest) and the *Erico-Pinion* (basophilic subcontinental pine forest) alliances (Delarze et al. 2015; Käsermann and Moser 1999), suggesting that woodland type is not so constraining as previously thought. Concerning soil properties, it has been shown that *C. calceolus* can grow on a large variety of soil types (Kļaviņa and Osvalde 2017; Rusconi et al. 2022) – with some caveats. First, *C. calceolus* appears to grow better in base-rich soils containing calcium carbonates (Kull 1999), which usually occur over limestone or dolomite bedrock (Käsermann and Moser 1999; Kull 1999). On the one hand, according to Käsermann and Moser (1999) soil pH requirements of this species range from neutral to moderately acidic. On the other hand, Rusconi et al. (2022) described pH requirements from neutral to alkaline. Moreover, *C. calceolus* seems to prefer richer substrates when in the shade compared to when growing in sunnier conditions, which may be due to the competition effect with other plant species (Käsermann and Moser 1999). Indeed, Kļaviņa and Osvalde (2017) noticed a positive relationship between the population size of *C. calceolus* and soil organic matter concentration. Finally, Devillers-Terschuren (1999) showed that *C. calceolus* grow better on moderately moist soils. In short, while theory suggests that a narrow window of biotic and abiotic factors is required for *C. calceolus* to thrive, the ecological requirements for this species tend to be broad, widespread, and context-dependent making the selection of (re)introduction sites, at least at first sight, very complex and hard to predict.

With this study we aimed to determine the relationships between the performance of *C. calceolus* populations and soil and vegetation parameters, in order to improve this species conservation and but also to advance the theoretical underpinning of which facets of an ecosystem most influence a species fitness. To this end, we characterised the vegetation communities and edaphic properties in 34 Swiss populations of *C. calceolus* (17 small and 17 large populations) and measured functional traits related to plant growth and reproduction. Specifically, we assumed that several unique functional traits are indicators of plant fitness and population health, as highlighted by Adler et al. (2014). Specifically, we asked: 1) which plant functional traits best describe differences between small and large populations? 2) Is there a specific or recurrent pattern in vegetation composition or/and structure in relation to *C. calceolus* presence and population size? 3) Are there combinations of companion plant species that specifically dictate *C. calceolus* population size? 4) Is the population size of *C. calceolus* dependent on local edaphic properties? We hypothesised that while this plant species has broad ecological preferences, some facets of the vegetation or soil physico-chemical properties can be used to explain why some populations perform better than others (Devillers-Terschuren 1999; Kļaviņa and Osvalde 2017; Kull 1999; Onuch and Skwaryło-Bednarz 2014), and therefore, plant species performance, estimated using specific plant functional traits, could be linked to unique combinations of vegetation and soil properties, which ultimately would be used to inform more efficient conservation efforts.

## Material and methods

### Population selection criteria

**Fig. 1:**
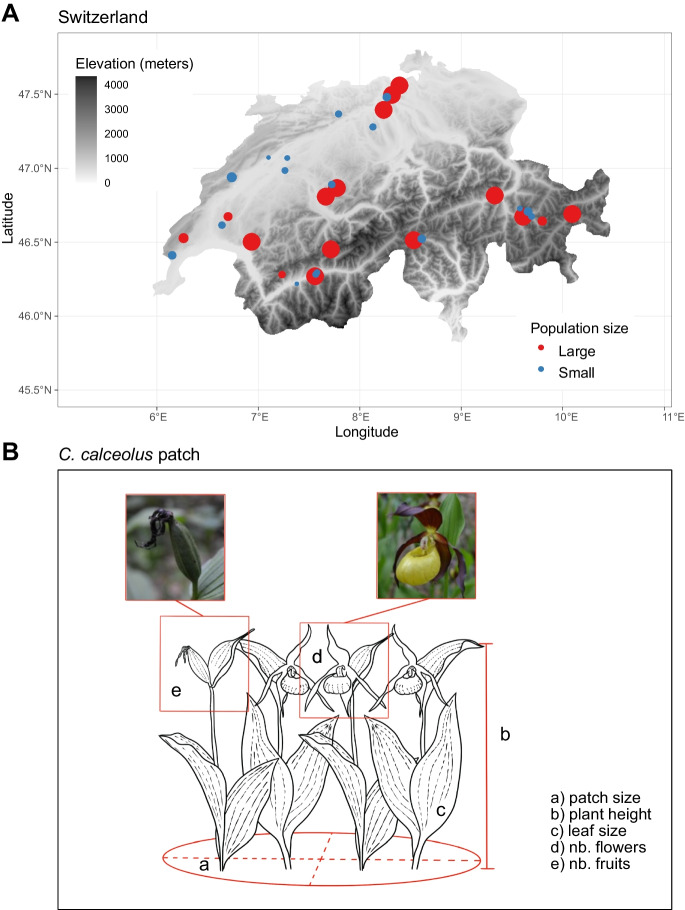
Sampling design. Shown are (**A**) an elevation map of Switzerland including the 34 populations of *C calceolus* surveyed during this study. Dots are coloured based on the population being small (blue dots; < 10 individuals), or large (red dots; > 20 individuals). The size of the dots is scaled according to the clone patch size of each *C. calceolus* individual. The average percent cover of the *C. calceolus* populations in the survey vegetation patches were 6.6%, and 3.1% for large and small populations, respectively (Figure S1). **B** Plant traits measured on *C. calceolus* individuals: a) clonal patch area calculated as an ellipse, b) plant height taken from the soil to the top of the highest leave (foliaceous bract) on the highest stem of the patch, c) leaf area calculated on the median leaf using the ellipse formula on the highest stem of the patch, d) number of flowers per patch and e) number of fruits (pods) per patch. Sampling of flowers and fruits were done at two time points, separated in average by a period of about two months. Other measures on the plants, but not shown here include number of leaves, leaf photosynthetic activity, specific leaf area (SLA), number of stems per patch and fruit volume

We chose *C. calceolus* populations of varying sizes from throughout Switzerland based on known occurrences of viable populations (data provided by www.infoflora.ch). We selected a total of 34 populations, 17 small (i.e., less than 10 individuals per population) and 17 large (i.e., more than 20 individuals per population) (Figure S1), encompassing the six major biogeographic regions of Switzerland (Fig. 1A). Each population was visited between 2017 and 2021, once during the peak flowering period, between April and June, and once after the production of fruits (i.e., seed pods), between July and August.

### Vegetation and plant traits

During the first visit to each *C. calceolus* population, we performed a comprehensive vegetation survey on a 10 × 10 m^2^ plot comprehending the most homogenous vegetation type of each site following the Braun-Blanquet method (Braun-Blanquet 1964). We used measured vegetation community structure (characteristic and most abundant species) to assign an alliance name to each vegetation type based on phytosociological nomenclature (Barkman et al. 1986), and according to Delarze et al. (2015) (Table 1). We next measured ten functional traits with known relationships with plant life history parameters (Adler et al. 2014; Díaz et al. 2016; Pérez-Harguindeguy et al. 2016) that could be quantified using non-intrusive methods. At each site, we sampled traits on a maximum of 10 randomly chosen individuals growing within a 100 m^2^ area and separated by a minimum of 2 m from each other (Fig. 1B). Each stem or group of stems (patch), separated by a minimum of 70 cm from each other, was considered as an individual (i.e., a clonal patch; Fig. 1B) (Kull 1999). For growth-related traits, we measured clonal patch area (cm^2^), the vegetative plant height (cm), leaf number and median leaf area (cm^2^, ellipse area formula) of the highest stem in a clonal patch and the median leaf photosynthetic activity (SPAD chlorophyllometer, Konica Minolta, Osaka Japan) and specific leaf area (SLA, mm^2^/mg) of 1 leaf per patch. For reproductive traits, we counted the number of stems and the number of flowers per patch on the first visit, and on the second visit counted, per patch, the number fruits (pods) and estimated median fruit volume (approximated as a cylinder; Fig. 1B).

**Table 1:** Soil and vegetation types measured across 34 populations of *Cypripedium. calceolus.* Columns, from left to right, correspond to the name of the 34 sites as shown in Figure S2 and 6. Site names are abbreviations of the canton where each population stands and the site number (Aargau: AG, Bern: BE, BL: Basel-Landschaft, GR: Graubünden, NE: Neuchâtel, VD: Vaud, VS: Valais and TI: Ticino). The two-level factor of populations being either small (< 10 individuals), or large (> 20 individuals), the elevation in meters, the names of the soil types according to IUSS Working Group (2015), the Latin name of the vegetation alliance at each site (according to (Delarze et al. 2015)), the corresponding vegetation type, the most abundance plant species at each site, and the number of species found at each site

Site	Size	Elevation	Soil_type	Alliance	Vegetation type	Dominant species	Richness
AG1	Small	501	Calcaric Cambisol (Colluvic)	*Galio-Fagenion*	Low altitude mesophyll beech forest	*Petasites albus*	39
AG2	Small	600	Calcaric Cambisol	*Galio-Fagenion*	Low altitude mesophyll beech forest	*Acer pseudoplatanus*	44
AG3	Large	450	Calcaric Cambisol	*Cephalanthero-Fagenion*	Xerothermophile beech forest	*Brachypodium sylvaticum*	47
AG4	Large	467	Calcaric Cambisol	*Galio-Fagenion*	Low altitude mesophyll beech forest	*Brachypodium sylvaticum*	48
AG5	Large	564	Calcaric Cambisol (Colluvic)	*Galio-Fagenion*	Low altitude mesophyll beech forest	*Frangula alnus*	60
BE1	Small	440	Calcaric Cambisol	*Cephalanthero-Fagenion*	Xerothermophile beech forest	*Corylus avellana*	34
BE2	Small	606	Calcaric Cambisol	*Cephalanthero-Fagenion*	Xerothermophile beech forest	*Fraxinus excelsior*	16
BE3	Small	500	Calcaric Cambisol (Colluvic)	*Cephalanthero-Fagenion*	Xerothermophile beech forest	*Fagus sylvatica*	37
BE4	Small	870	Calcaric Cambisol (Colluvic)	*Lonicero-Fagenion*	Beech forest of lower montane level	*Picea abies*	36
BE5	Large	780	Calcaric Cambisol	*Lonicero-Fagenion*	Beech forest of lower montane level	*Brachypodium sylvaticum*	41
BE6	Large	915	Calcaric Cambisol (Colluvic)	*Lonicero-Fagenion*	Beech forest of lower montane level	*Bromus benekenii*	39
BE7	Large	1540	Calcaric Cambisol (Colluvic, Humic)	*Vaccinio-Piceion*	Spruce forest	*Calamagrostis varia*	49
BL1	Small	933	Rendzic Calcaric Leptosol (Colluvic)	*Abieti-Fagenion*	Beech-fir forest of montane level	*Picea abies*	17
GR1	Small	1923	Dolomitic Cambisol	*Erico-Pinion unicinatae*	Mountain pine forest	*Larix decidua*	35
GR2	Small	1128	Gypsiric Dolomitic Cambisol	*Cephalanthero-Fagenion*	Xerothermophile beech forest	*Carex alba*	44
GR3	Small	1915	Gypsiric Dolomitic Cambiosol	*Erico-Pinion sylvestris*	Basophilic subcontinental pine forest	*Pinus mugo*	37
GR4	Large	880	Calcaric Cambisol (Humic)	*Cephalanthero-Fagenion*	Xerothermophile beech forest	*Picea abies*	31
GR5	Small	1700	Gypsiric Dolomitic Cambisol	*Vaccinio-Piceion*	Spruce forest	*Deschampsia cespitosa*	40
GR6	Large	1527	Dolomitic Cambisol	*Vaccinio-Piceion*	Spruce forest	*Larix decidua*	41
GR7	Large	1153	Calcaric Cambisol (Colluvic)	*Erico-Pinion sylvestris*	Basophilic subcontinental pine forest	*Picea abies*	26
NE1	Small	1166	Calcaric Cambisol (Colluvic)	*Lonicero-Fagenion*	Beech forest of lower montane level	*Carex sempervirens*	32
NE2	Small	1187	Rendzic Calcaric Leptosol (Colluvic)	*Abieti-Fagenion*	Beech-fir forest of montane level	*Calamagrostis varia*	29
TI1	Small	1265	Dolomitic Cambisol	*Sambuco-Salicion*	Pre-forest shrub stage	*Calamagrostis varia*	51
TI2	Large	1348	Dolomitic Cambisol	*Vaccinio-Piceion*	Spruce forest	*Brachipodium pinatum*	52
VD1	Small	658	Calcaric Cambisol (Colluvic)	*Galio-Fagenion*	Low altitude mesophyll beech forest	*Carex flacca*	29
VD2	Small	669	Calcaric Cambisol (Humic)	*Molinio-Pinion*	Subatlantic pine forest on marl slopes	*Acer platanoides*	43
VD3	Large	994	Calcaric Cambisol	*Abieti-Fagenion*	Beech-fir forest of montane level	*Calamagrostis varia*	35
VD4	Large	1117	Calcaric Cambisol	*Abieti-Fagenion*	Beech-fir forest of montane level	*Hordelymus europaeus*	48
VD5	Large	621	Rendzic Calcaric Leptosol	*Galio-Fagenion*	Low altitude mesophyll beech forest	*Picea abies*	34
VS1	Small	731	Leptic Calcaric Cambisol	*Cephalanthero-Fagenion*	Xerothermophile beech forest	*Sesleria albicans*	34
VS2	Small	571	Stagnic Calcaric Cambisol	*Cephalanthero-Fagenion*	Xerothermophile beech forest	*Pinus sylvestris*	29
VS3	Small	1075	Calcaric Cambisol	*Abieti-Piceion*	Spruce-fir forest	*Salix caprea*	47
VS4	Large	1040	Calcaric Leptosol (Colluvic)	*Cephalanthero-Fagenion*	Xerothermophile beech forest	*Betula pendula*	25
VS5	Large	1450	Calcaric Cambisol	*Erico-Pinion unicinatae*	Mountain pine forest	*Erica carnea*	46

### Site and soil parameters

During the second visit, in each site, we dug a soil profile from the surface to the bedrock or parental material at a minimum distance of 40 cm from the nearest plant. We identified organic, organo-mineral and mineral horizons to characterise the soil type (Baize and Girard 2009; IUSS Working Group 2015) and corresponding humus forms (Zanella et al. 2018). Then, within the 100 m^2^ area of each site, we sampled approximately 300 g of the organo-mineral horizon (A) three times, corresponding to a depth of 20.9 ± 8.5 cm – i.e., the rooting zone of *C. calceolus*. From the A horizons we measured eight soil physicochemical parameters, including; 1) relative humidity (HR), obtained by subsequent desiccation at 105 °C and weighing. 2) Total organic carbon (Corg) to total nitrogen (Ntot), and subsequent carbon-to-nitrogen ratio (CN), which were measured using an elemental analyzer (Flash 2000, CHN-O Analyzer, Thermo Scientific, Waltham, Massachusetts, United States). 3) Total soil organic matter content (SOM), measured via the loss of ignition (LOI) method, by heating the samples at 450 °C for two hours. 4) pH, measured in distilled water with a Metrohm 827 pH meter (Metrohm AG, Herisa, Switzerland). 5) Soil total cationic exchange capacity (CEC), using the “cobalt hexamine trichloride” method (Ciesielski and Sterckeman 1997). 6) Total carbonates, estimated by CaCO_3_ dissolution after HCl 6 M addition, and according to the Calcimeter Bernard method (Dreimanis 1962). 7) Bioavailable phosphorus (P) estimated by extraction with sodium bicarbonate (Olsen 1954). 8) Nitrate (NO_3_^−^) quantified after an extraction with potassium sulphate (Bremner and Shaw 1955). In addition, on soils that developed on a dolomitic substrate (geological information obtained from on www.mapgeo.admin.ch), we performed dolomite quantification using the Calcimeter Bernard method (Dreimanis 1962) and gypsum quantification using a method with successive weighing after a passage in the desiccator and in the oven (105 °C) (Lebron et al. 2009).

### Statistical analyses

All analyses were performed in R (R Core Development Team 2021).

#### *Cypripedium calceolus* population functional traits

We performed a principal component analysis (PCA) to visualize the multivariate trait space of all *C. calceolus* populations (*dudi.pca* function in the package *ade4*, (Dray and Dufour 2007)). We next performed a Regularized Discriminant Analysis (RDA) with the *rda* function in the package *vegan* (Oksanen et al. 2013), and a one-way ANOVA on the first axis of the PCA to assess the effect of population size (two levels; small and large) on *C. calceolus* functional traits. Site was included as a blocking factor in the analyses.

#### Vegetation community relationship with* C. calceolus* population size and functional traits

**Fig. 2:**
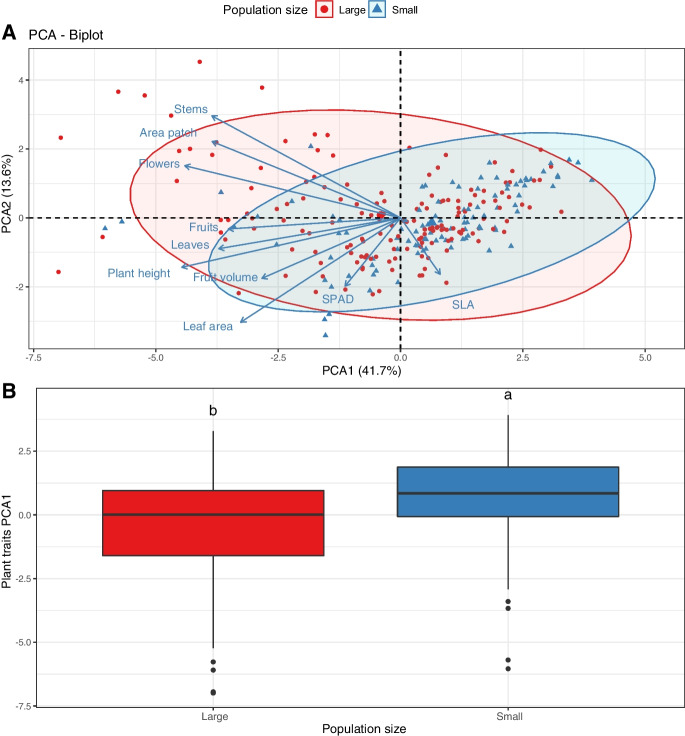
Functional traits characterizing *Cypripedium calceolus* population size. **A** Principal component analysis (PCA) of seven plant traits describing the functional form of the different *C. calceolus* populations (see methods for details on the traits). Ellipses show the 95% confidence intervals characterizing small (blue area; < 10 individuals), and large (red areas, > 20 individuals) populations. **B** Boxplots describing average values of small (blue) and large (red) based on the first axis of the PCA as shown above. Letters above boxplots show significant differences among populations (TukeyHSD, p < 0.05)

First, we performed a clustering analysis on the entire vegetation matrix to potentially detect clusters of similar vegetation types that best explain *C. calceolus* population size and plant traits. For this, we calculated a distance matrix of the vegetation communities using the *vegdist* function in *vegan* (Bray–Curtis distance) and then calculated the optimal number of groups using pairwise dissimilarities (distances) between communities in the vegetation data set (*daisy* function metric = “gower” in the *cluster* package (Maechler et al. 2013)). To assess a potential correlation between vegetation structure and the *C. calceolus* functional traits we performed a Mantel test between the trait distance matrix (Euclidean distance) and the obtained vegetation distance matrix (Bray–Curtis distance) (*mantel.test* function in *vegan*). Using a phylogenetic signal approach, we also calculated a potential correlation between dendrogram branch length and values for the number of fruits (a trait most representative of *C. calceolus* fitness) and the first principal component (PC1; as shown in Fig. 2) using the *phyloSignal* function in the package *phylobase* (Hackathon 2020). Next, to assess the effect of *C. calceolus* population size (two levels; small and large) on the plant community structure of each site, we used a permutational ANOVA (PERMANOVA), using the *adonis* function in the *vegan* package. The Bray–Curtis metric was used to calculate dissimilarity among vegetation communities. Finally, independently of the vegetation community structure, we assessed potential indicator species associated with large or small *C. calceolus* populations species using the *multipatt* function in the *indicspecies* package (De Caceres et al. 2016).

#### Soil properties across site and relationship with* C. calceolus* population size and functional traits

First, we classified soil profiles following two nomenclatures, the Référentiel pédologique classification (Baize and Girard 2009)), and the world soil reference database WRB (IUSS Working Group 2015). Humus forms were classified according to Zanella et al. (2018). Next, we performed a principal component analysis (PCA) to visualize the multivariate space of soil properties for all *C. calceolus* populations (*dudi.pca* function in the package *ade4* (Dray and Dufour 2007)). We performed a Regularized Discriminant Analysis (RDA) with the *rda* function in the package *vegan* (Oksanen et al. 2013), and a multivariate analysis of variance (MANOVA) to assess the effect of population size (two levels; small and large) on the soil properties of *C. calceolus* populations. Site was included as a blocking factor in the analyses. Second, we assessed the potential correlation between individual soil properties and the *C. calceolus* population functional trait matrix using redundancy analysis (*rda* function) and the *ordistep* function (package *vegan*) to perform a backward selection of variables in the model.

## Results

**Fig. 3:**
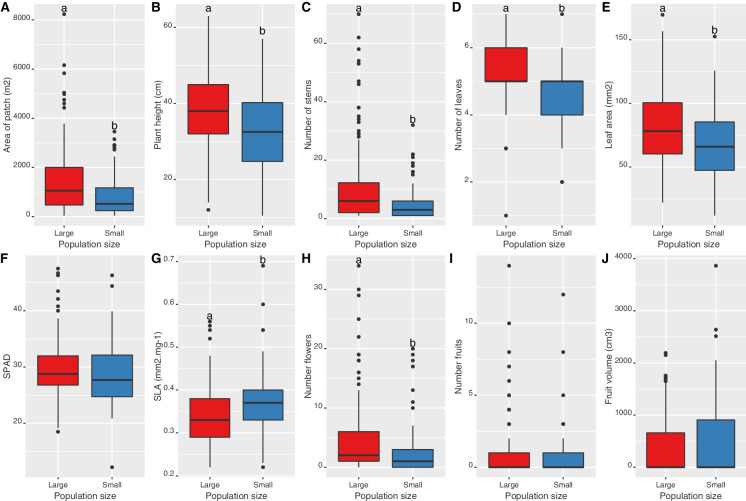
* Cypripedium calceolus* population functional traits. Shown are average trait values distributions for A) individual patch area (approximated to an ellipse) B) plant height, C) number of stems per patch, D) number of leaves per plant, E) leaf area, F) chlorophyll content (SPAD), G) specific leaf area (SLA), H) number of flowers per plant, I) number of fruits per plant, J) fruit volume (approximated to a cylinder) for small (blue boxplots; < 10 individuals), and large (red boxplots, > 100 individuals) populations. Different letters above boxplots show significant differences (p < 0.05) based on the multivariate analysis of variance as shown in Table S1

C. calceolus *population functional traits* – We found that large populations of *C. calceolus* contained on average 72% bigger patches with 2.5 times more stems, 18% bigger plants, 5.5% more leaves, and 20% bigger and 7% harder leaves, and produced 83% more flower per individual than small populations (Fig. 3, Table S1). Overall, multivariate trait space was significantly separated by the categorical variable of population size (Fig. 2A, RDA with 999; F_1,221_ = 4.39, p = 0.001), and accordingly, along the first axis of the PCA (Fig. 2B, population size treatment effect; F_1,221_ = 24.62, p < 0.001, and site effect; F_32,221_ = 4.65, p < 0.001).

### Vegetation community relationships with *C. calceolus* population size and functional traits

Based on the vegetation surveys, we found that *C. calceolus* populations were associated with 349 vascular plant species in total and found in 10 different vegetation alliances (Table 1), with surrounding plant species richness ranging from 17 species to a maximum of 60 species per site (Table 1). The dominant species varied strongly between sites, with a total of 34 different plant species being dominant when considering all sites (Table 1). Overall, we found that the best clustering model identified 12 groups, suggesting a high variability in vegetation types across sites (Figure S2). Accordingly, we did not find a correlation between the population functional trait matrix and the vegetation community structure (Mantel test on 10,000 permutations, r = 0.14, z = 53.95, p = 0.14, Figure S2). Similarly, the population size had no effect on the surrounding vegetation community structure (PERMANOVA, F_1,33_ = 0.97, p = 0.48). These results were also supported by a generally weak dendrogram distance-based signal (k and lambda values) for all traits (see k and lambda values in Table S2 indicating a weak correlation between the trait values and the branch length of the dendrogram; i.e., more similar vegetation types do not correspond to more similar traits values; p > 0.05).

The indicator species analysis highlighted that the most discriminant species based on *C. Calceolus* population size were *Ajuga reptans* (r = 0.64, p = 0.014), *Juniperus communis* (r = 0.56, p = 0.019), *Equisetum telmateia* (r = 0.50, p = 0.037), and *Gymnadenia conopsea* (r = 0.50, p = 0.037). Indeed, *A. reptans* was 19 times more abundant (glm with quasipoisson distribution with Type II analysis of deviance, Chi-sq = 9.41, p = 0.002), *J. communis was* 1.7 times more abundant (Chi-sq = 12.56, p = 0.003), *E. telmateia* was in average present with a relative abundance of 2.11% in large populations, while virtually absent in small populations (Chi-sq = 7.83, p = 0.005), and *G. conopsea* was 12 times more abundant (Chi-sq = 9.02, p = 0.003) in large *C. calceolus* populations than in small populations. We also found that small population of *C. calceolus* were significantly associated to two species; *Sesleria caerulea* (Chi-sq = 0.68, p = 0.031), and *Carex sempervirens* (Chi-sq = 0.53, p = 0.044).

### Soil properties across site and relationship with *C. calceolus* population size and functional traits

**Fig. 4:**
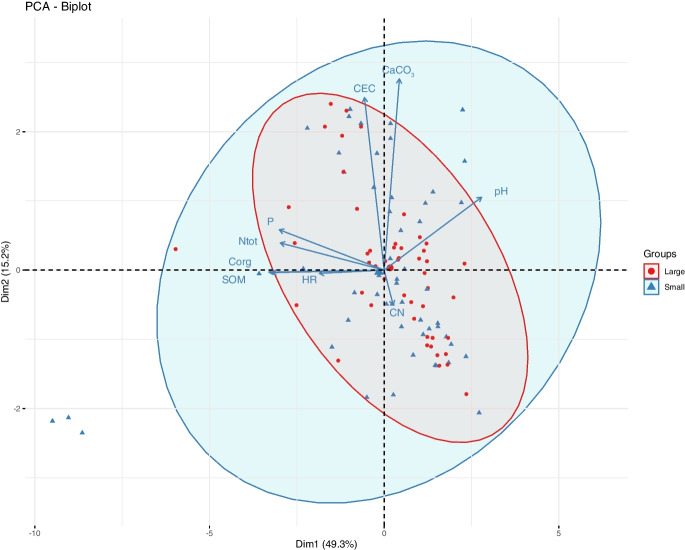
Principal component analysis (PCA) of ten soil physico-chemical traits describing the soils form of the 34 different *Cypripedium calceolus* populations. Ellipses show the 95% confidence intervals characterizing small (blue area; < 10 individuals), and large (red areas, > 20 individuals) populations. Soil traits included: soil relative humidity, pH, total soil organic matter content, CaCO_3_, Total N, total C, G) carbon-to-nitrogen ratio (CN), cation exchange capacity (CEC), total phosphorous (P), and total nitrates (NO_3_)

**Fig. 5:**
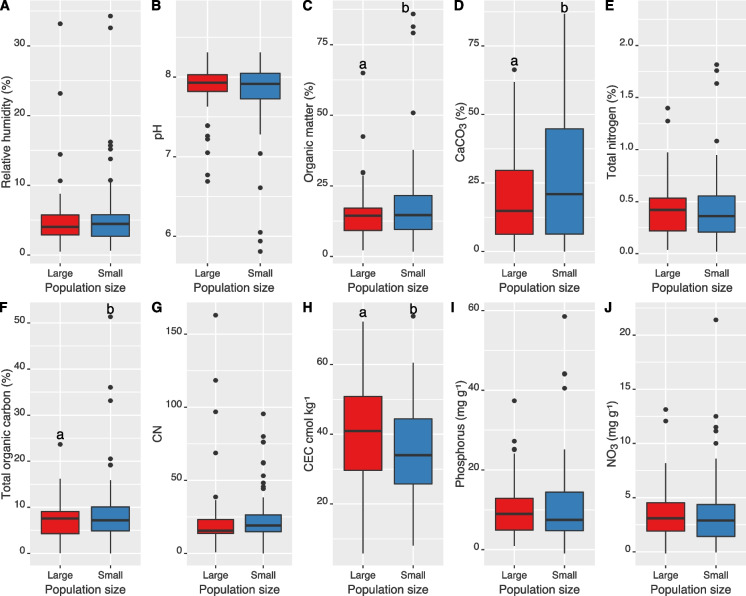
Soil physico-chemical parameters of *Cypripedium calceolus* populations. Shown are average trait values distributions for A) soil relative humidity (HR) B) pH, C) total soil organic matter content (SOM), D) CaCO_3_, E) Total N, F) total C, G) carbon-to-nitrogen ratio (CN), H) cation exchange capacity (CEC), I) total phosphorous, and J) total nitrates (NO_3_) for small (blue boxplots; < 10 individuals), and large (red boxplots, > 20 individuals) populations. Different letters above boxplots show significant differences (p < 0.05) based on the multivariate analysis of variance as shown in Table S3

**Fig. 6:**
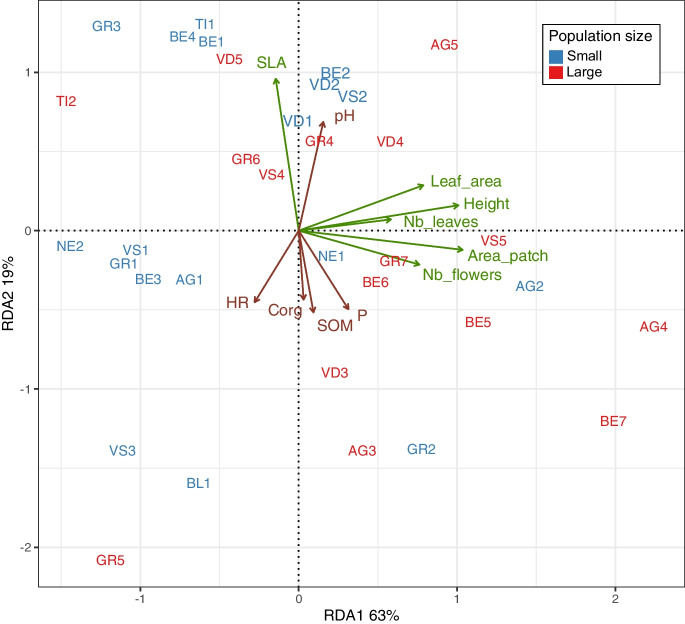
Correlation between plant functional traits and soil physico-chemical parameters. Shown is a Regularized Discriminant Analysis (RDA) biplot for highlighting the relative importance of soil physicochemical parameters (brown arrows) on the functional traits (green arrows) of *C calceolus* populations. Site names are coloured based on the categorical variable population size (small = blue colour; < 10 individuals, and large = red colour, > 20 individuals). Only those soil variables that are significantly correlated with the vegetation functional trait matrix are shown with brown arrows (p < 0.05)

Overall, we identified three major soil classes according to Baize and Girard (2009), all being calcaric or dolomitic soils: 23 Calcosols (Calcaric Cambisol (IUSS Working Group 2015)), four Rendosols (Calcaric Leptosols, (IUSS Working Group 2015)), and seven Dolomitosols (Dolomitic Cambisols, (IUSS Working Group 2015) (Table 1 and Supplementary data D1). Humus forms ranged between Mull and Moder (Supplementary data D1). We found that small and large populations significantly differed in soil physicochemical properties, with small populations inhabiting a wider range of variance than large populations (Fig. 4; RDA with 999; F_33,69_ = 6.53, p = 0.001). Considering all traits together also revealed clear differences among sites (Fig. 5, MANOVA, population size effect; Pillai = 0.68, approximation_10,60_ = 12.98, p < 0.001, site effect; Pillai = 6.3, approximation_320,6900_ = 3.67, p < 0.001). Specifically, we found that sites occupied by small populations had soil containing 21% more SOM, 26% more Corg, and 40% more CaCO_3_, but 16% less CEC than soil beneath large populations of *C. calceolus* (Fig. 5 and Table S3). Through the RDA analysis and stepwise model selection, we found that the soil characteristics that best explain *C. calceolus* population functional traits were pH (F_1,60_ = 2.54, p = 0.05), HR (F_1,60_ = 2.61, p = 0.06°), P (F_1,60_ = 3.37, p = 0.03), SOM (F_1,60_ = 3.78, p = 0.02), and Corg (F_1,60_ = 4.25, p = 0.02) (Fig. 6). In other words, we observed that high values of pH (alkaline soils) correlated positively with leaf area and SLA. In addition, P, SOM and Corg were positively correlated with the patch area and the number of flowers, while high HR values were negatively correlated with population fitness traits (Fig. 6).

## Discussion

Through extensive and fine-grained field work we explored the links between functional traits related to population size and vegetation and soil parameters. We found that large (> 20 individuals) populations of *C. calceolus* display a specific assemblage of measurable characteristics that discriminate them from small (< 10 individuals) populations, indicating that it is possible to assess the health of a population of a rare plant species by measuring a specific set of functional traits. While we could not highlight a direct link between vegetation alliances and soil types and population size, we show that the unique combination of companion plants, and several edaphic parameters, such as SOM, CaCO_3_, pH, and P, could be used to potentially assess the optimal sites to implement (re)-introduction actions for this emblematic and patrimonial orchid species.

### Functional trait variation across small and large populations

We found that, based on our classification, small and large populations display different functional signatures, in which, large populations of *C. calceolus* displayed significantly higher values for most measured plant traits, compared to small populations. This indicates that classic plant functional traits (Díaz et al. 2016; Wright et al. 2004) could be used to characterize the size of *C. calceolus* populations (Adler et al. 2014). In plant evolutionary ecology and conservation, the predicted relationship between plant population size and fitness has been amply observed (Reed 2005). For instance, Leimu et al. (2006) performed a meta-analysis on 105 studies focusing on correlations between plant population size, fitness and genetic variation. They highlighted that all these correlations were significantly positive. Moreover, rather interestingly for our study, the same research also showed that these positive relationships were more pronounced for rare than for common species, an effect thus likely heightened when large population size differences exist in nature. Relationships between populations size and fitness may happen for two reasons: (i) an extinction vortex causing a decrease in genetic variation leading to inbreeding depression (Ruegg and Turbek 2022), a reduced mate availability or random genetic drift that consequently reduce populations fitness; or (ii) a difference in habitat quality (Ellstrand and Elam 1993; Fischer and Matthies 1998; Leimu et al. 2006). From a conservation point of view, these hypotheses are even more central in the case of an endangered species such as *C. calceolus*. Indeed, small populations, which can be crucial for a species survival, are more vulnerable to stochastic events and fluctuations (Honnay and Jacquemyn 2007; Reed 2005). The recovery time after a perturbation is lengthened by a reduced fitness and will make the population more prone to extinction when supplementary perturbations happen (Reed 2005). In fact, smaller populations appear to be less able to adapt to new environmental changes because of the loss of adaptative genetic variation through genetic drift (Reed et al. 2003; Willi et al. 2007). In addition, reduced pollinator activity in small populations of rare species generally contributes to reduced plant fitness (Leimu et al. 2006). For the *C. calceolus* populations studied here, further investigations should be made to understand whether the relationship between population size and fitness is due to inbreeding depression or to habitat quality. Genetic studies performed in Europe on *C. calceolus* populations showed that this species has a relatively high genetic diversity compared with rare taxa and taxa with the same life history (Brzosko 2002; Brzosko et al. 2002). On the other hand, signatures of a bottleneck effect and recent founder events were identified in Estonia, and genetic and genotypic diversity variables were significantly correlated with population size in Poland (Brzosko et al. 2011; Gargiulo et al. 2018). Finally, in addition to genetic studies and for conservation purposes, it would be necessary to understand the minimum size of *C. calceolus* populations so as not to be threatened by the extinction vortex and then ensure that populations stay under this threshold.

### Vegetation communities associated with *C. calceolus*

Across our sampling, we found that *C. calceolus* grows on 11 vegetation alliances, with those where *C. calceolus* mostly occurred being xerothermophilic beech forests (*Cephalanthero-Fagenion*) and the low elevation mesophyll beech forest (*Galio-Fagenion*) – as observed by Käsermann and Moser (1999), but we couldn’t find a recurrent pattern between vegetation composition and *C. calceolus* population size. It is essential to highlight that vegetation alliances were difficult to assess because *C. calceolus* often grows in somewhat hybrid environments (i.e., transition zones) that do not always fit traditional classification methods (Delarze et al. 2015). Consequently, we were only able to associate our vegetation inventories with the closest vegetation alliances found in the literature (see Table 1). All identified vegetation alliances were forests, except for the pre-forest shrub stage (*Sambuco-Salicion*). Together, these findings indicate that *C. calceolus* prefer to grow at the forest edges, or in the ecotones, of mixed-stands forest type, therefore preferring intermediate levels of direct solar radiations (i.e., not in full light, but neither in full understory shading) (Rusconi et al. 2022). Along these lines, Hurskainen et al. (2017) highlighted that removing trees to create forest gaps favoured *C. calceolus* populations significantly. These observations point to the very complex ecology of this orchid and the delicate balance of light parameters it requires to grow (Kirillova and Kirillov 2019). Accordingly, because open forests are disappearing in Switzerland, recovery of *C. calceolus* populations might be impacted. In this regard, specific forest management plans should be implemented to favour this species optimal light requirements (Bornand et al. 2016). However, based on our findings, vegetation composition per se is not a sufficiently strong marker for the identification of suitable (re)introduction sites in Switzerland, and other parameters should be accounted for.

### The use of indicator species for finding suitable habitats

Through species indicator analyses, we found that large and small *C. calceolus* populations were best discriminated by four species, positively by *Ajuga reptans*, *Juniperus communis, Equisetum telmateia*, and *Gymnadenia conopsea,* while negatively *by Sesleria caerulea* and *Carex sempervirens.* Interestingly, the ecological characteristics of *A. reptans* (the most discriminant species for large populations of *C. calceolus*) reflect the ecological needs of *C. calceolus*: clear forests with average humidity and average soil nutrients (Landolt et al. 2010). However, *A. reptans* would not be a good indicator species for finding *C. calceolus* suitable habitats, as it is even more broadly distributed than *C. calceolus* in Switzerland, growing in the sub-alpine regions (Lauber et al. 2018). The three other species positively discriminating large populations also shared similar or close ecological requirements to *C. calceolus* and the same elevation optimum (Landolt et al. 2010). The two species discriminating small populations had the same soil nutrient requirements than *C. calceolus,* but their ecological optima are at higher elevations (Landolt et al. 2010). Therefore, a combination of some of the observed positively and negatively discriminating species could be used to find suitable habitats for *C. calceolus.* Such an approach could be confirmed using a combination of fieldwork for assessing population fitness (as was done here) and species distribution modelling (Guisan et al. 2006). It is important to highlight that these results could be regional and should not be generalized for the entire distribution range of *C. calceolus* without further investigations (Devillers-Terschuren 1999).

### Soil characteristics associated with *C. calceolus* population size

Based on the soil horizon profile analysis, we observed that *C. calceolus* grows on three, or two depending on the soil nomenclature, soil types, which corresponds to the observations made by Käsermann and Moser (1999). In opposition with the results of the vegetation types, and based on the total number of existing soil references (110 in Baize and Girard (2009) and 32 soil groups in IUSS (IUSS Working Group 2015)), we thus found that *C. calceolus* grows on a very limited range of soil types, attesting that *C. calceolus* preferentially grows on calcareous or dolomitic substrate (with average pH of 7.81). Moreover, the physicochemical characteristics results corroborate what is generally found in the literature: *C. calceolus* grows in soil with neutral to alkaline pH (Rusconi et al. 2022) with the presence of calcium carbonates (in the form of CaCO_3_ or CaMg(CO_3_)_2_) (Käsermann and Moser 1999) and with on average a high concentration of soil organic matter (Kļaviņa and Osvalde 2017). The observations regarding the humus forms, Mull-to-Moder, also support the notion that the rooting of this plant is where biological activity is relatively intense and where the organic matter is well and rapidly integrated into the soil matrix (Zanella et al. 2018). Moreover, through multivariate comparative analysis, we found that soil parameters that most strongly influenced *C. calceolus* functional traits were pH, HR, P, Corg and SOM. In this regard, our results contradict those found by Kļaviņa and Osvalde (2017), in which pH did not affect population vitality. A precise characterization of the edaphic niche of endangered species (as we did in this study) is crucial for implementing conservation plans and identifying suitable (re)introduction sites. Specifically for *C. calceolus*, we encourage to perform soil physicochemical analysis to verify that the preselected zone has the following edaphic properties: presence of CaCO_3_ or CaMg(CO_3_)_2_, neutral to alkaline pH, about 15% of organic matter and a CEC of about 40 cmol/kg.

That said, while we mostly focused on measuring the importance of physicochemical properties, we acknowledge that we did not take in account soil organisms, such as the orchid-associated mycorrhizal fungi (Sathiyadash et al. 2012). Indeed, the general dogma is that because orchids have dust-like seeds (0.3–14 μg), with minimal nutrients reserves, their interaction with orchid mycorrhizae (OM) is vital for the plant to overcome the first steps of germination and development (e.g., Sathiyadash et al. 2012; Smith and Read 2010). Accordingly, the rarity of some orchid species could be linked to the sparse distribution or narrow ecological requirements of their OM partners (e.g., Fay et al. 2015; Nurfadilah et al. 2013; Phillips et al. 2011). To date, however, broad-level ecological knowledge of OM associated with *C. calceolus* is largely lacking (Fay et al. 2018), and future work should address the soil community versus root community relationship, and the characterization of unique OM strains associated to well-performing orchid populations.

In conclusion, with this work, we provide an additional step toward a better understanding how the selection of (re)introduction sites for the conservation of endangered plant species can be resolved. Specifically, we argue that beyond the use of classic approaches for site selection, such as building species distribution models (Guisan et al. 2013; Pecchi et al. 2019; Prasad et al. 2016), or, on the opposite, using only practitioner-informed current or past occurrence knowledge (Rusconi et al. 2022), might not provide the most accurate predictions for finding optimal sites to enhance or protect the target species. Instead, a fine-grained analysis of multiple targeted ecosystem variables is generally needed (Prasad et al. 2016; Richardson et al. 2009). Moreover, the analyses derived from our fieldwork also highlighted a direct link between plant and population life history traits and in situ measurable functional traits. In return, we then highlighted that narrow ranges of edaphic factors best correlated with unique sets of the measured plant traits, ultimately corroborating the importance of above-belowground links in plant ecology (Wardle 2002), and conservation biology.

## Supplementary Information

Below is the link to the electronic supplementary material.

Supplementary file1 (DOCX 20811 KB)

## Data Availability

Because of the sensitive nature of this dataset linked to an endangered species, the datasets generated during and/or analysed during the current study are available from the corresponding author on reasonable request.
