# Supplementary material for: Soil properties and plant species can predict population size and potential introduction sites of the endangered orchid Cypripedium calceolus
Source: Plant Soil. 2023 Feb 16;487(1-2):467–83. doi: 10.1007/s11104-023-05945-4 (PMC10272267; doi:10.1007/s11104-023-05945-4)
Supplement: Supplementary file 1 — Supplementary file1 (DOCX 20811 KB) [file 11104_2023_5945_MOESM1_ESM.docx]

**Supplementary material for** *Plant and Soil*

Soil properties and plant species can predict population health and potential introduction sites of the endangered orchid *Cypripedium calceolus*

Olivia Rusconi^1^*, Théo Steiner^1^, Claire Le Bayon^1^ and Sergio Rasmann^1^*

^1^ Institute of biology, University of Neuchâtel, Rue Emile-Argand 11, 2000 Neuchâtel.

* correspondence - OR: olivia.rusconi@unine.ch; SR: sergio.rasmann@unine.ch, phone: +41 332 718 23 37

**Table S1.** Multivariate analysis of variance (MANOVA) results table for all plant functional traits measured across 34 *C. calceolus* populations.

| Responsevariable | Factor | Df | Pillai | Fnum/Fden | Pr(>F) |  |
| --- | --- | --- | --- | --- | --- | --- |
| All_traits | Pop_size | 1 | 0.3066 | 10/212 | <0.001 | *** |
|  | Site | 32 | 3.2981 | 320/2210 | <0.001 | *** |
|  | Residuals | 221 |  |  |  |  |
|  |  | Df | SumSq | F | Pr(>F) |  |
| Plant size | Pop_size | 1 | 2079.2 | 27.22 | <0.001 | *** |
|  | Site | 32 | 10833.7 | 4.43 | <0.001 | *** |
|  | Residuals | 221 | 16880.2 |  |  |  |
| Nb of Flower | Pop_size | 1 | 303.6 | 12.72 | <0.001 | *** |
|  | Site | 32 | 2728.9 | 3.57 | <0.001 | *** |
|  | Residuals | 221 | 5275.4 |  |  |  |
| Nb of fruits | Pop_size | 1 | 7.06 | 2.45 | 0.12 |  |
|  | Site | 32 | 213.13 | 2.31 | 0.00 | *** |
|  | Residuals | 221 | 637.55 |  |  |  |
| Fruit volume | Pop_size | 1 | 462777 | 1.38 | 0.24 |  |
|  | Site | 32 | 35980689 | 3.36 | <0.001 | *** |
|  | Residuals | 221 | 74011715 |  |  |  |
| Nb of stems | Pop_size | 1 | 1951.9 | 28.15 | <0.001 | *** |
|  | Site | 32 | 14595.7 | 6.58 | <0.001 | *** |
|  | Residuals | 221 | 15323.2 |  |  |  |
| Nb of leaves | Pop_size | 1 | 4.202 | 5.93 | 0.02 | * |
|  | Site | 32 | 79.452 | 3.50 | <0.001 | *** |
|  | Residuals | 221 | 156.683 |  |  |  |
| Leaf area | Pop_size | 1 | 11048 | 18.80 | <0.001 | *** |
|  | Site | 32 | 86449 | 4.60 | <0.001 | *** |
|  | Residuals | 221 | 129845 |  |  |  |
| SPAD | Pop_size | 1 | 40.9 | 1.87 | 0.17 |  |
|  | Site | 32 | 2356.8 | 3.36 | <0.001 | *** |
|  | Residuals | 221 | 4848 |  |  |  |
| SLA | Pop_size | 1 | 0.04262 | 19.04 | <0.001 | *** |
|  | Site | 32 | 0.78676 | 10.98 | <0.001 | *** |
|  | Residuals | 221 | 0.4948 |  |  |  |
| Area patch | Pop_size | 1 | 21941923 | 22.38 | <0.001 | *** |
|  | Site | 32 | 132072274 | 4.21 | <0.001 | *** |
|  | Residuals | 221 | 216707442 |  |  |  |

Signif. codes: 0 ‘***’ 0.001 ‘**’ 0.01 ‘*’ 0.05 ‘.’ 0.1 ‘ ’ 1

**Table S2**. Phylogenetic signal type of analyses to detect potential association between dendrogram branch length (as shown in Figure S2) and trait values across 34 populations of *C. calceolus.* Signals are calculated both with K and lambda statistics. The associated p-values indicate potential deviation from null values, if p < 0.05.

| Variable | K | P (K) | Lambda (λ) | P (λ) |
| --- | --- | --- | --- | --- |
| Patch area | 0.59 | 0.11 | 0.00 | 1.00 |
| Plant height | 0.56 | 0.20 | 0.05 | 0.71 |
| Nb stems | 0.56 | 0.25 | 0.00 | 1.00 |
| Nb leaves | 0.55 | 0.22 | 0.04 | 0.75 |
| Leaf area | 0.56 | 0.20 | 0.14 | 0.27 |
| SPAD | 0.58 | 0.13 | 0.00 | 1.00 |
| SLA | 0.56 | 0.19 | 0.00 | 1.00 |
| Nb flowers | 0.56 | 0.19 | 0.00 | 1.00 |
| Nb fruits | 0.48 | 0.70 | 0.00 | 1.00 |
| Fruit size | 0.49 | 0.62 | 0.00 | 1.00 |

**Table S3**. Multivariate analysis of variance (MANOVA) results table for all soil physico-chemical properties measured across 34 *C. calceolus* populations.

| Response variable | Factor | Df | Pillai | Fnum/Fden | Pr(>F) |  |
| --- | --- | --- | --- | --- | --- | --- |
| All_traits | Pop_size | 1 | 0.68 | 10/60 | <0.001 | *** |
|  | Site | 32 | 3.67 | 320/690 | <0.001 | *** |
|  | Residuals | 69 |  |  |  |  |
|  |  | Df | SumSq | F-value | Pf(>F) |  |
| HR | Pop_size | 1 | 10.7 | 0.55 | 0.462 |  |
|  | Site | 32 | 2370.3 | 3.79 | <0.001 | *** |
|  | Residuals | 69 | 1349.8 |  |  |  |
| SOM | Pop_size | 1 | 294.4 | 6.17 | 0.015 | * |
|  | Site | 32 | 19554.6 | 12.81 | <0.001 | *** |
|  | Residuals | 69 | 3292 |  |  |  |
| CaCO3 | Pop_size | 1 | 1722 | 21.76 | <0.001 | *** |
|  | Site | 32 | 45210 | 17.85 | <0.001 | *** |
|  | Residuals | 69 | 5461 |  |  |  |
| pH | Pop_size | 1 | 0.1631 | 2.40 | 0.126 |  |
|  | Site | 32 | 16.3437 | 7.52 | <0.001 | *** |
|  | Residuals | 69 | 4.6879 |  |  |  |
| P | Pop_size | 1 | 16.5 | 0.59 | 0.444 |  |
|  | Site | 32 | 8121.7 | 9.14 | <0.001 | *** |
|  | Residuals | 69 | 1916.4 |  |  |  |
| CEC | Pop_size | 1 | 1024.4 | 13.19 | 0.001 | *** |
|  | Site | 32 | 15773.2 | 6.34 | <0.001 | *** |
|  | Residuals | 69 | 5360.7 |  |  |  |
| Ntot | Pop_size | 1 | 0.0133 | 0.40 | 0.531 |  |
|  | Site | 32 | 10.5278 | 9.84 | <0.001 | *** |
|  | Residuals | 69 | 2.306 |  |  |  |
| CN | Pop_size | 1 | 78 | 0.22 | 0.642 |  |
|  | Site | 32 | 37225 | 3.27 | <0.001 | *** |
|  | Residuals | 69 | 24526 |  |  |  |
| NO3 | Pop_size | 1 | 1.88 | 0.34 | 0.560 |  |
|  | Site | 32 | 719.12 | 4.10 | <0.001 | *** |
|  | Residuals | 69 | 378.13 |  |  |  |
| Corg | Pop_size | 1 | 96.846 | 7.5121 | 0.008 | ** |
|  | Site | 32 | 128.293 | 9.9514 | <0.001 | *** |
|  | Residuals | 69 | 12.892 |  |  |  |
|  |  |  |  |  |  |  |

**Figure S1**. **Average percent cover of *C. calceolus* populations in the surveyed vegetation patches according to the factorial discrimination of population size** (Small = blue boxplots, and Large = red boxplots). Letters above boxplots indicate significant differences (glm with quasipoisson distributin, t_1,29_ = -3.202, p = 0.003).

**Figure S2.** **Similarities across C. calceolus-associated vegetation communities.** Shown is a dendrogram based on Bray-Curtis dissimilarities values of plant species occurrences across 34 sites. Each site is coloured based on the size of C. calceolus population (small = blue colour; < 10 individuals, and large = red colour, > 20 individuals). Adjacent to the dendrogram are shown the scaled and cantered values for all functional vegetation traits in each population, as shown in Figure 4.

**Supplementary data D1. Soil profiles of 34 *Cypripedium calceolus* populations.** In each *C. calceolus* population, we dug a soil profile until bedrock or parental material, and we separated soil profiles in organic, organo-mineral and mineral horizons according to each homogenous zone observed. We identified each horizon and classified both the soils and humus forms (Baize and Girard 2009; IUSS Working Group 2015; Zanella et al. 2018). The humus and soil horizons were named according to the Référentiel pédologique (Baize and Girard 2009), and the World Reference Base for Soil Resources (WRB) (IUSS Working Group 2015) (see glossary of soil terms at the end of the document. Finally, vegetation types were also identified and classified according to Delarze et al. (2015).

Glossary for humus forms and soil horizons according to (Zanella et al. 2018) with (IUSS Working Group 2015) equivalent.

| **Horizon name** | **Definition** | **WRB** |
| --- | --- | --- |
| nOL and vOL | The OL horizon is characterized by the accumulation of mainly leaves/needles, twigs and woody materials, most of the original plant organs being easily discernible to the naked eye. The humic component amounts to less than 10% by volume; recognizable remains 90% and more, up to 100% in non-decomposed litter. Suffixes characterize neither fragmented nor transformed/discolored leaves and/or needles (nOL), and slightly altered, discolored, bleached, softened up, glued, matted, skeletonized, sometimes only slightly fragmented leaves and/or needles (vOL) | O |
| BryoOL | OL horizon with Bryo characteristics defined as an horizon with “byophytes or arbuscular lichens or small stonecrop plants totally covering the soil and forming a stratified carpet or cushion with living (green) parts overgrowing a layer of dead stems and leaves | O |
| OF | The OF horizon is characterized by the accumulation of partly decomposed litter, mainly from transformed leaves/needles, twigs and woody materials, but without any entire plant organ. The proportion of humic component is 10% to 70% by volume. Depending on humus form, decomposition is mainly accomplished by soil fauna (OFzo) or cellulose–lignin decomposing fungi (OFnoz). Slow decomposition is revealed by a partly decomposed matted layer, permeated by hyphae | O |
| OH | The OH horizon is characterized by an accumulation of zoogenically transformed material, i.e. black, gray-brown, brown, reddish-brown well-decomposed litter, mainly comprised of aged animal droppings. A large part of the original structures and materials are not discernible, the humic component amounting to more than 70% by volume. The OH horizon differs from the OF horizon by a more advanced transformation (fragmentation, humification, ...) due to the action of soil organisms | O |

Soil horizons and layers according to (Baize and Girard 2009) with (IUSS Working Group 2015) equivalent.

| **Horizon name** | **Definition** | **WRB** |
| --- | --- | --- |
| A | A horizon presents a mix of organic and mineral material and is situated under holorganic horizons. | A |
| Aca | Aca is an A horizon with cold HCl effervescence. | A |
| Acaho | Acaho is an Aca horizon that is humus-rich. | A |
| Aci | Aci is an Aca horizon which has been decarbonated. Aci is thus not carbonated in fine soil or or only occasionally or locally. | A |
| Ado | Ado is an A horizon which is made up of hard dolomitic rocks. | A |
| Adoy | Adoy is an A horizon which is made up of hard dolomitic rocks and contains gypsum. | A |
| Agca | Agca is an Aca horizon with cold HCl effervescence and with redoxic properties. |  |
| Cca | C horizon is a depth mineral horizon. Its components have undergone, throughout their mass, significant fragmentation and/or some geochemical alteration. Cca is calcareous C horizon. | C |
| Cdo | Cdo is an C horizon which is made up of hard dolomitic rocks. | C |
| Mca | M layer is formed by continuous, loose or soft rocks, not or slightly fragmented, with possibly locally or partially altered micro-cracks. This layer are coherent, but easy to work with tools. Mca is calcareous M layer. | - |
| S | S horizon is a structural horizon. It is an alteration horizon. Process such as alteration of primary minerals, release of iron oxyhydroxides, decarbonation take place in that horizon. | B |
| Sca | Sca is calcareous S horizon. | B |
| Sdo | Sdo is an S horizon which is made up of hard dolomitic rocks. | B |
| Sdoy | Sdoy is an S horizon which is made up of hard dolomitic rocks and contains gypsum. | B |
| Sgca | Sgca is an Sca horizon with cold HCl effervescence and with redoxic properties. | B |

| **Soil classification** | | | |
| --- | --- | --- | --- |
| **Soil name (Référentiel pédologique, 2009)** | | | CALCOSOL colluvial, issu d'éboulis calcaires |
| **Soil name (WRB, 2015)** | | | Calcaric Cambisol (Colluvic) |
| **Humus form (Zanella et al., 2018)** | | | Eumull |
| **Horizon sequence (Référentiel pédologique, 2009)** | | | [OL]-OF-BryoOL / Aca / Sca-Aca / Sca-[Aca] |
| **Site name** | | | AG1 |
|  | | | |
| **Station parameters** | | | |
| **Location** | Ramsel Hang | 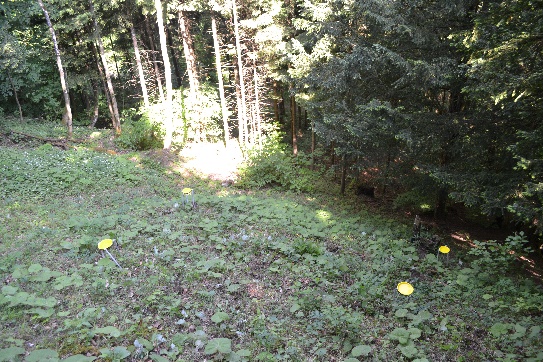 | |
| **Year of sampling** | 2018 |  |  |
| **Altitude** | 501 m |  |  |
| **Slope** | 40° |  |  |
| **Aspect** | 330° |  |  |
| **Topography** | Steep slope |  |  |
| **Geology** | Calcareous scree |  |  |
| **Vegetation type** | *Galio-Fagenion* |  |  |
|  | | | |
| **Soil profile** | | | |
| 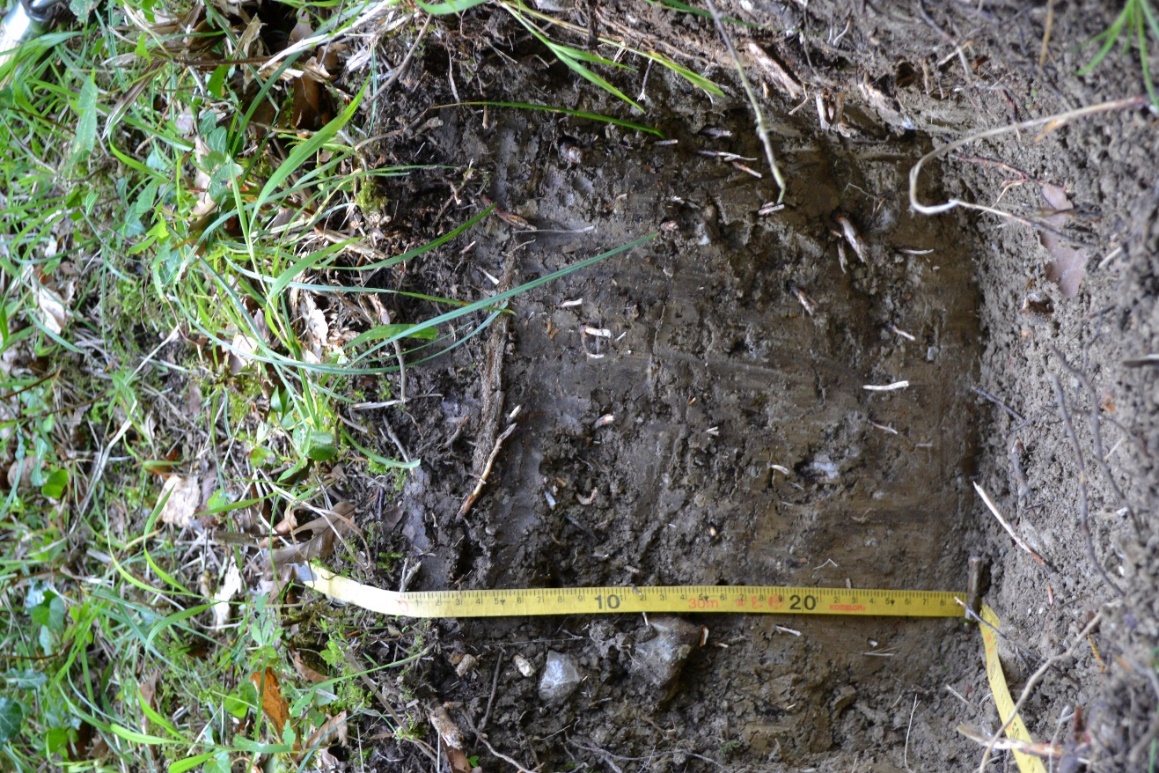 | | | |

| **Soil classification** | | | |
| --- | --- | --- | --- |
| **Soil name (Référentiel pédologique, 2009)** | | | CALCOSOL issu de molasse |
| **Soil name (WRB, 2015)** | | | Calcaric Cambisol |
| **Humus form (Zanella et al., 2018)** | | | Eumull |
| **Horizon sequence (Référentiel pédologique, 2009)** | | | [OL]-BryoOL / Aca / Aca-Sca / Sca |
| **Site name** | | | AG2 |
|  | | | |
| **Station parameters** | | | |
| **Location** | Erli/Bööndler | 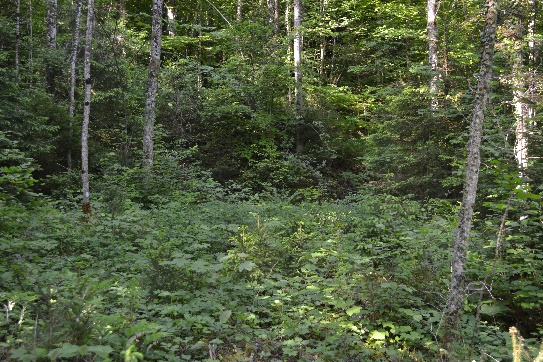 | |
| **Year of sampling** | 2018 |  |  |
| **Altitude** | 600 m |  |  |
| **Slope** | 8° |  |  |
| **Aspect** | 0° |  |  |
| **Topography** | Light north-facing slope |  |  |
| **Geology** | Molasse |  |  |
| **Vegetation type** | *Galio-Fagenion* |  |  |
|  | | | |
| **Soil profile** | | | |
| 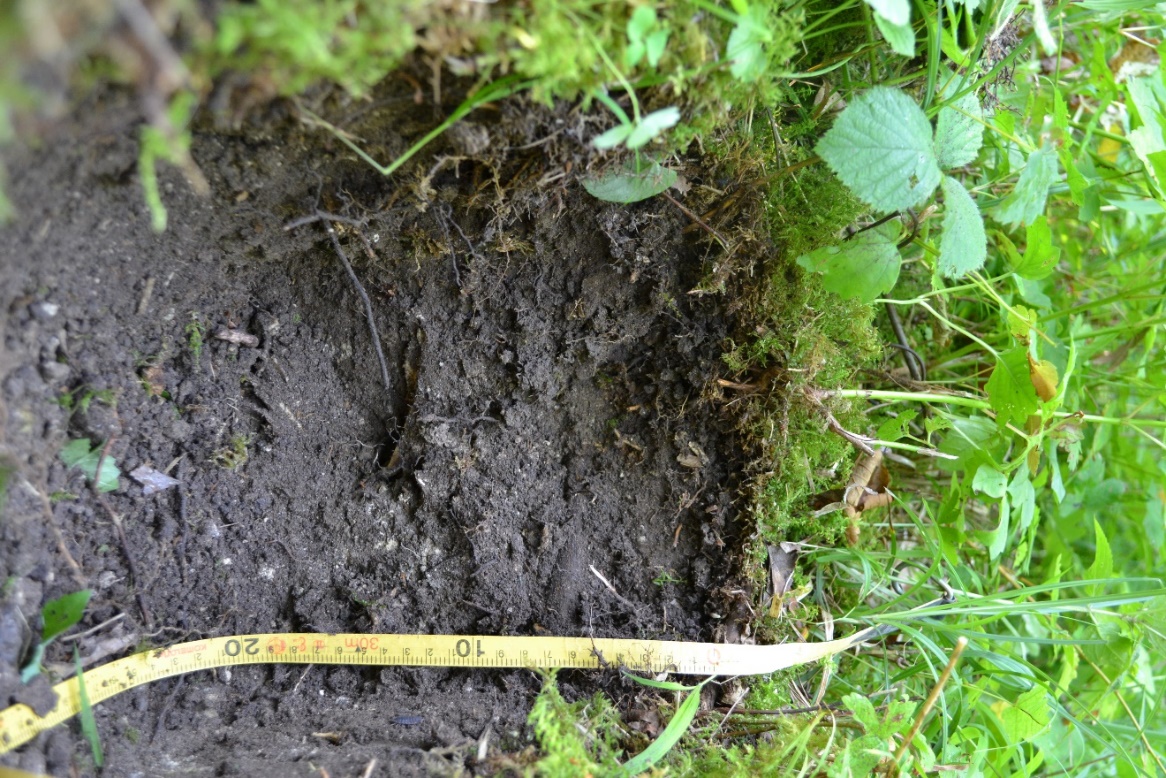 | | | |

| **Soil classification** | | | |
| --- | --- | --- | --- |
| **Soil name (Référentiel pédologique, 2009)** | | | CALCOSOL issu de molasse |
| **Soil name (WRB, 2015)** | | | Calcaric Cambisol |
| **Humus form (Zanella et al., 2018)** | | | Eumull |
| **Horizon sequence (Référentiel pédologique, 2009)** | | | [OL] / BryoOL-OF-Aca / Aca / Sca1 / Sca2 |
| **Site name** | | | AG3 |
|  | | | |
| **Station parameters** | | | |
| **Location** | Grashalde | 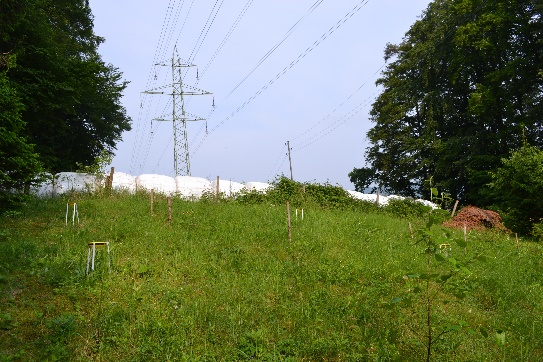 | |
| **Year of sampling** | 2018 |  |  |
| **Altitude** | 450 m |  |  |
| **Slope** | 10° |  |  |
| **Aspect** | 47° |  |  |
| **Topography** | Light slope |  |  |
| **Geology** | Molasse |  |  |
| **Vegetation type** | *Cephalanthero-Fagenion* |  |  |
|  | | | |
| **Soil profile** | | | |
| 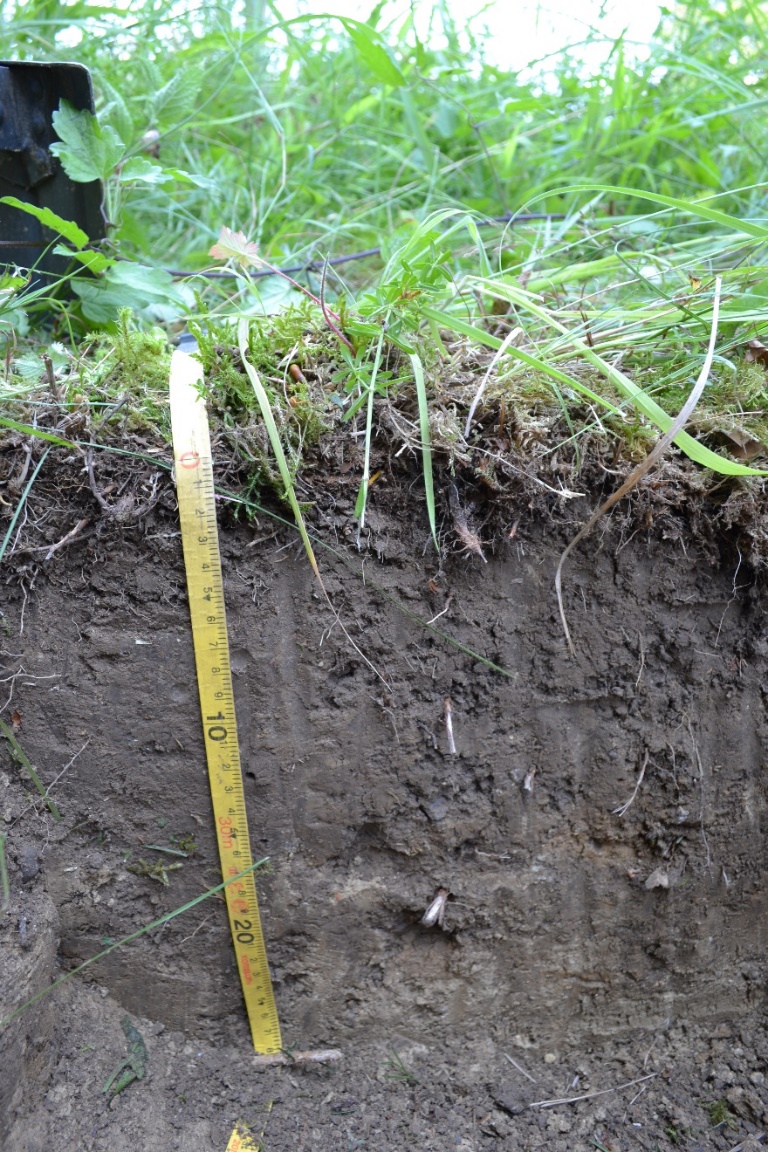 | | | |

| **Soil classification** | | | |
| --- | --- | --- | --- |
| **Soil name (Référentiel pédologique, 2009)** | | | CALCOSOL issu de molasse |
| **Soil name (WRB, 2015)** | | | Calcaric Cambisol |
| **Humus form (Zanella et al., 2018)** | | | Eumull |
| **Horizon sequence (Référentiel pédologique, 2009)** | | | [nOL-vOL] / Aca-Sca / Aca-Sca&Cca |
| **Site name** | | | AG4 |
|  | | | |
| **Station parameters** | | | |
| **Location** | Rotebüel | 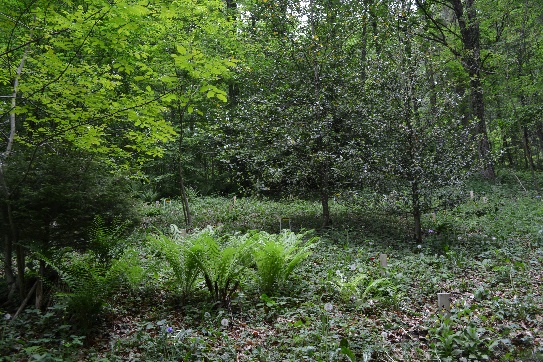 | |
| **Year of sampling** | 2018 |  |  |
| **Altitude** | 467 m |  |  |
| **Slope** | 10° |  |  |
| **Aspect** | 180° |  |  |
| **Topography** | Light slope |  |  |
| **Geology** | Molasse |  |  |
| **Vegetation type** | *Galio-Fagenion* |  |  |
|  | | | |
| **Soil profile** | | | |
| 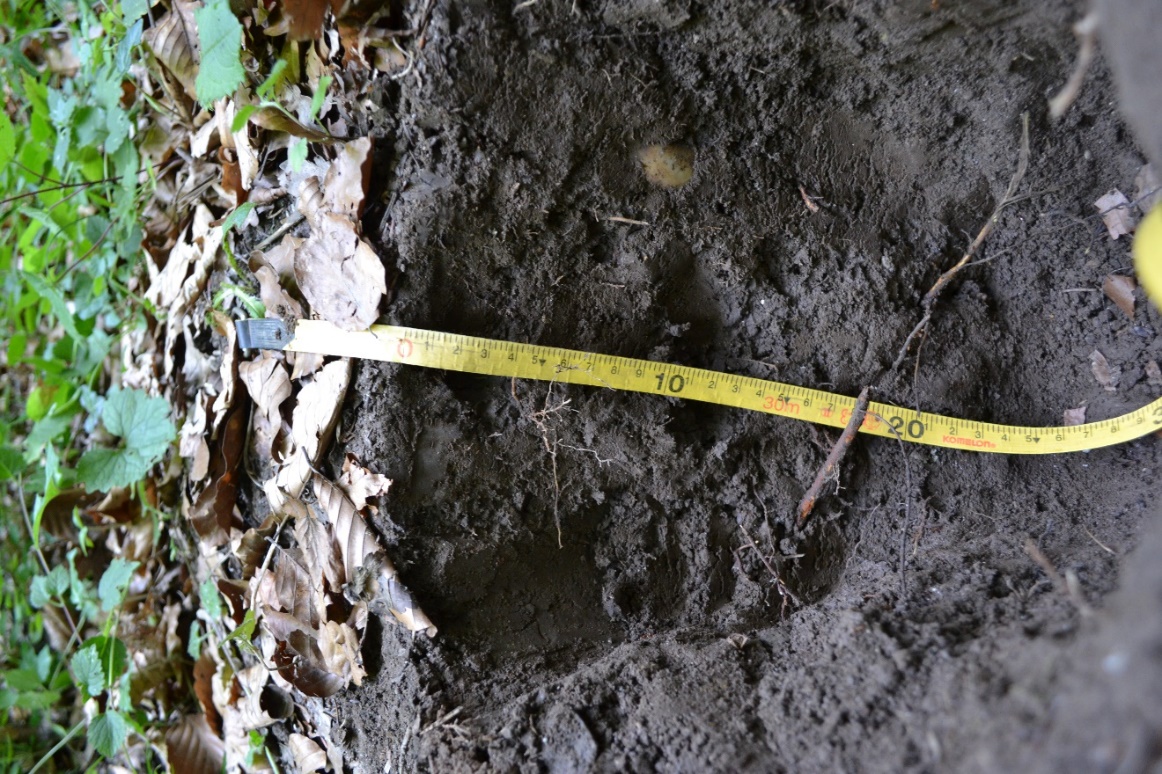 | | | |

| **Soil classification** | | | |
| --- | --- | --- | --- |
| **Soil name (Référentiel pédologique, 2009)** | | | CALCOSOL colluvial, issu d'éboulis calcaires |
| **Soil name (WRB, 2015)** | | | Calcaric Cambisol (Colluvic) |
| **Humus form (Zanella et al., 2018)** | | | Eumull |
| **Horizon sequence (Référentiel pédologique, 2009)** | | | [OL]-OF-BryoOL / Aca-Sca / Mca-[Aca-Sca] |
| **Site name** | | | AG5 |
|  | | | |
| **Station parameters** | | | |
| **Location** | Risi | 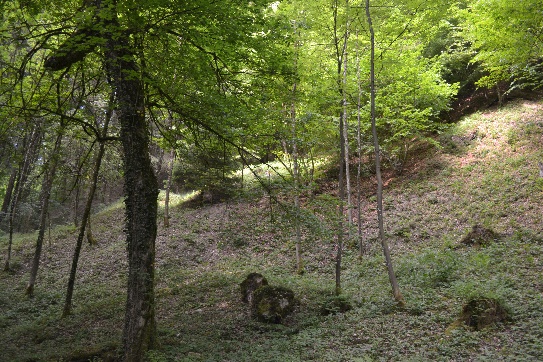 | |
| **Years of sampling** | 2016 and 2018 |  |  |
| **Altitude** | 564 m |  |  |
| **Slope** | 40° |  |  |
| **Aspect** | 90° |  |  |
| **Topography** | Steep slope |  |  |
| **Geology** | Calcareous scree |  |  |
| **Vegetation type** | *Galio-Fagenion* |  |  |
|  | | | |
| **Soil profile** | | | |
| 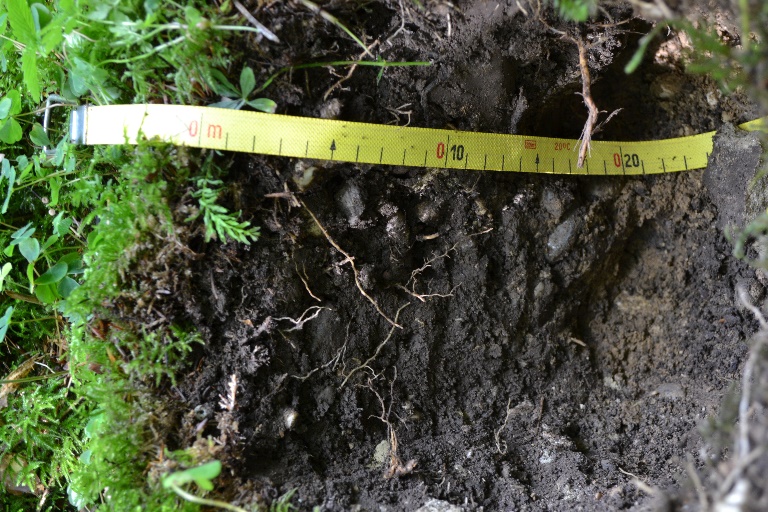 | | | |

| **Soil classification** | | | |
| --- | --- | --- | --- |
| **Soil name (Référentiel pédologique, 2009)** | | | CALCOSOL issu de molasse |
| **Soil name (WRB, 2015)** | | | Calcaric Cambisol |
| **Humus form (Zanella et al., 2018)** | | | Mesomull |
| **Horizon sequence (Référentiel pédologique, 2009)** | | | nOL-BryoOL / Aca / Sca / Sca-Mca / Mca |
| **Site name** | | | BE1 |
|  | | | |
| **Station parameters** | | | |
| **Location** | Chatzestiel | 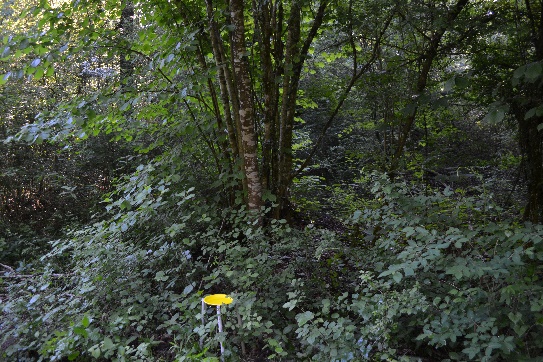 | |
| **Year of sampling** | 2018 |  |  |
| **Altitude** | 440 m |  |  |
| **Slope** | 0° |  |  |
| **Aspect** | 0° |  |  |
| **Topography** | Edge of a forest road, flat |  |  |
| **Geology** | Molasse |  |  |
| **Vegetation type** | *Cephalanthero-Fagenion* |  |  |
|  | | | |
| **Soil profile** | | | |
| 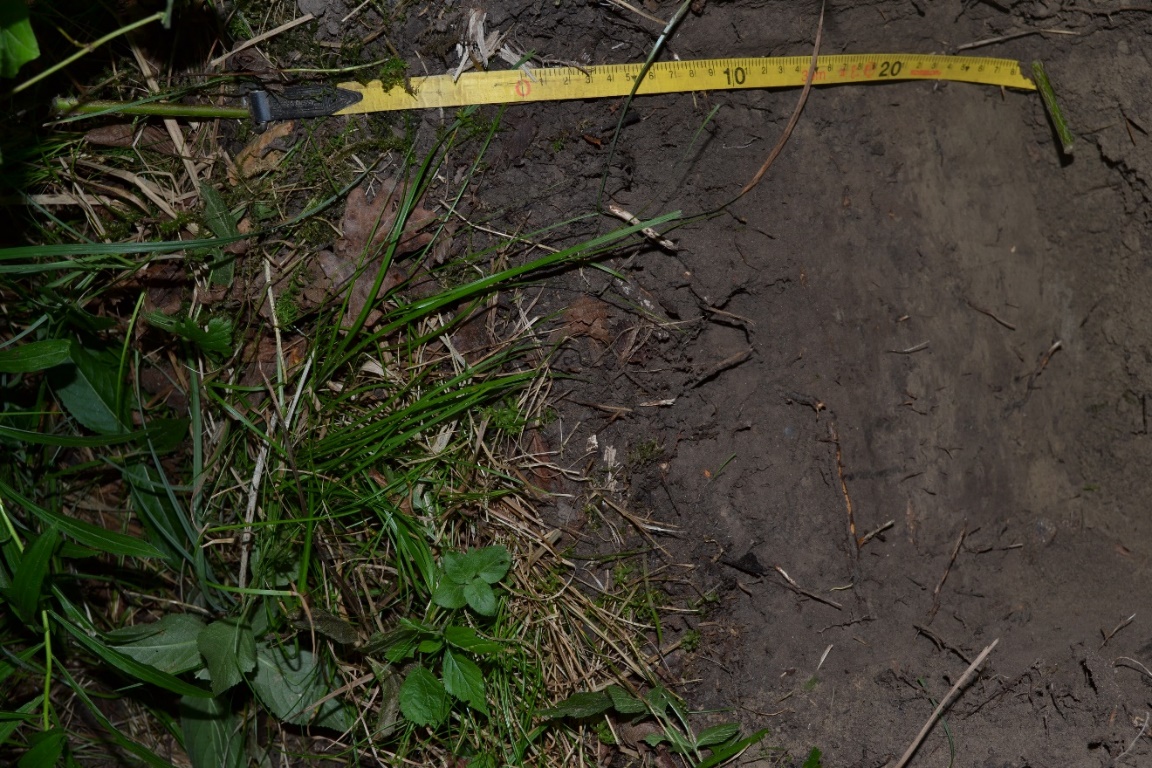 | | | |

| **Soil classification** | | | |
| --- | --- | --- | --- |
| **Soil name (Référentiel pédologique, 2009)** | | | CALCOSOL issu de calcaires du Portlandien |
| **Soil name (WRB, 2015)** | | | Calcaric Cambisol |
| **Humus form (Zanella et al., 2018)** | | | Dysmull |
| **Horizon sequence (Référentiel pédologique, 2009)** | | | nOL-vOL / OF-[Aca] / Aca-[OF] / Aca-Sca / Cca |
| **Site name** | | | BE2 |
|  | | | |
| **Station parameters** | | | |
| **Location** | La Neuveville | 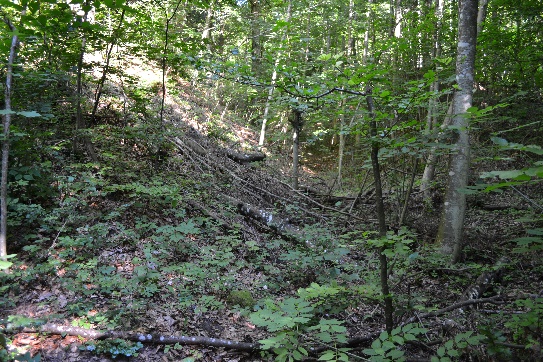 | |
| **Year of sampling** | 2016 and 2021 |  |  |
| **Altitude** | 606 m |  |  |
| **Slope** | 5° |  |  |
| **Aspect** | 68° |  |  |
| **Topography** | Concave bottom of slope |  |  |
| **Geology** | Portlandian limestone |  |  |
| **Vegetation type** | *Cephalanthero-Fagenion* |  |  |
|  | | | |
| **Soil profile** | | | |
| 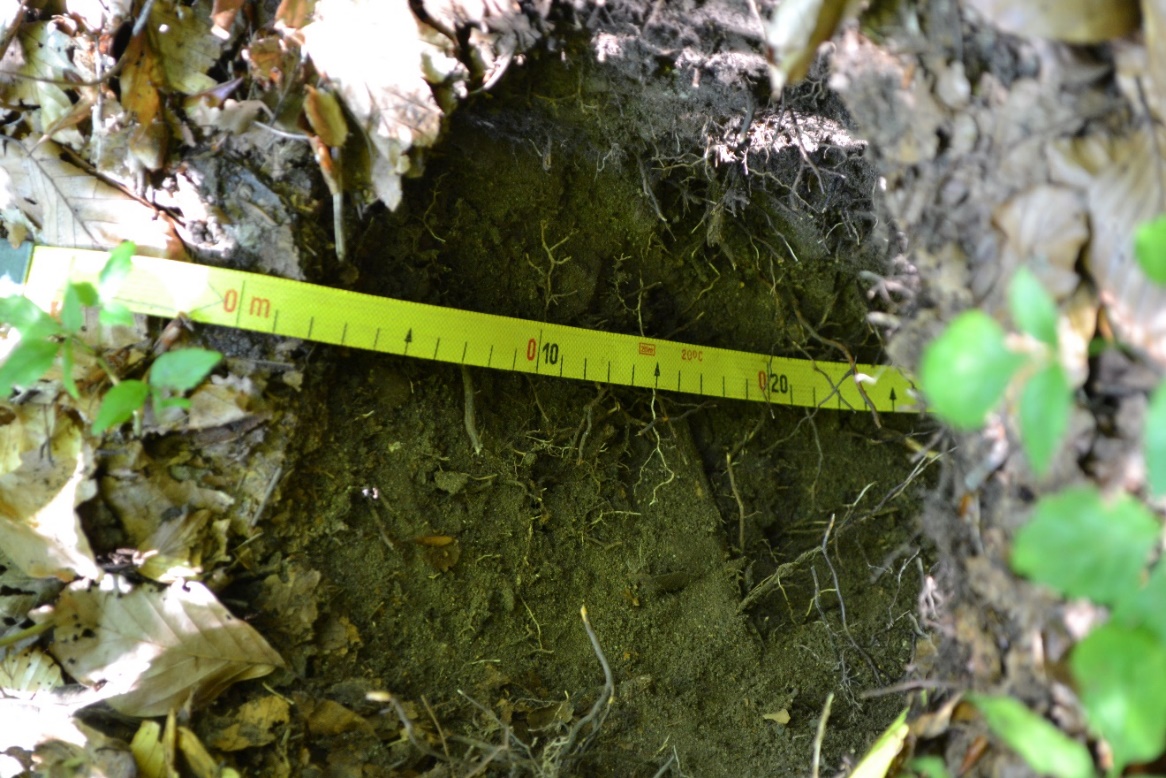 | | | |

| **Soil classification** | | |
| --- | --- | --- |
| **Soil name (Référentiel pédologique, 2009)** | | CALCOSOL colluvial, issu d’éboulis calcaires |
| **Soil name (WRB, 2015)** | | Calcaric Cambisol (Colluvic) |
| **Humus form (Zanella et al., 2018)** | | Mesomull |
| **Horizon sequence (Référentiel pédologique, 2009)** | | nOL-OF / Aca / Sca / Sca-Cca |
| **Site name** | | BE3 |
|  | | |
| 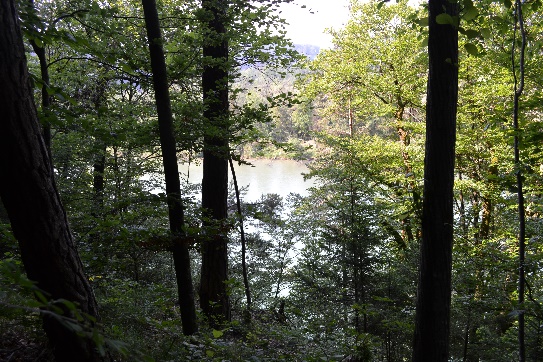**Station parameters** | | |
| **Location** | Schlosshubel |  |
| **Year of sampling** | 2016 and 2018 |  |
| **Altitude** | 500 m |  |
| **Slope** | 45° |  |
| **Aspect** | 220° |  |
| **Topography** | Steep slope under a molasse cliff |  |
| **Geology** | Molasse and calcareous scree |  |
| **Vegetation type** | *Cephalanthero-Fagenion* |  |
|  | | |
| **Soil profile** | | |
| 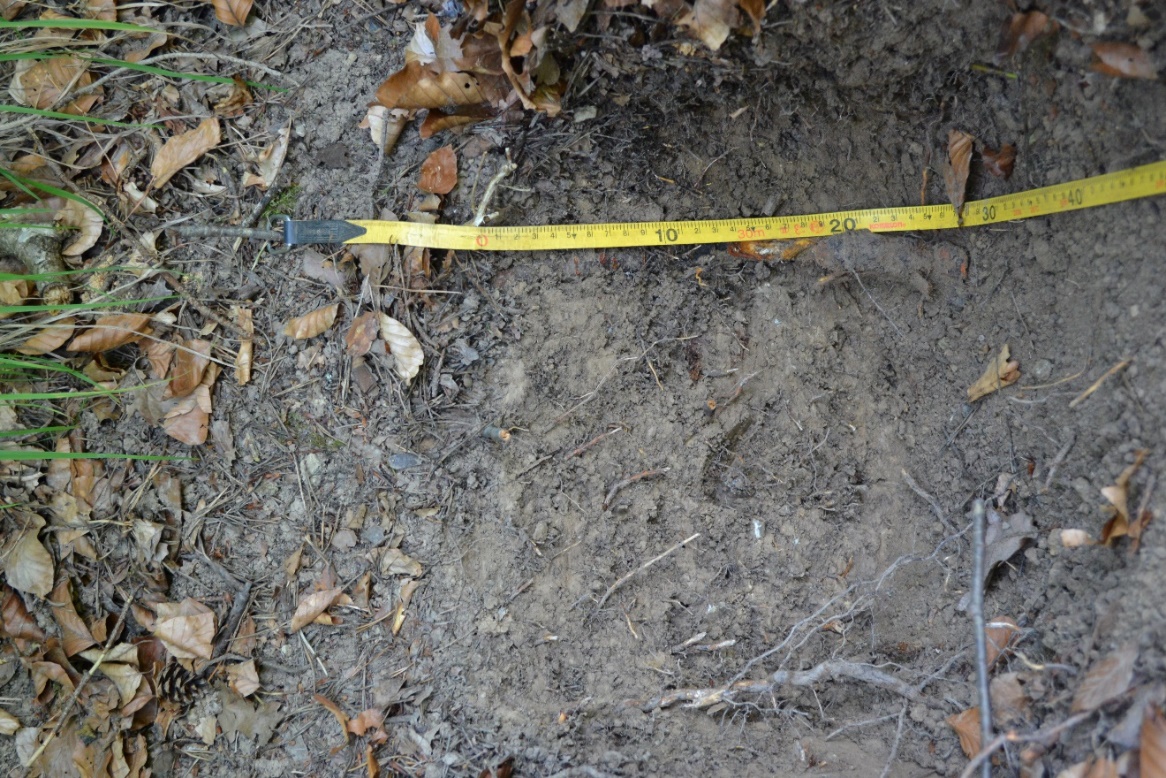 | | |

| **Soil classification** | | | |
| --- | --- | --- | --- |
| **Soil name (Référentiel pédologique, 2009)** | | | CALCOSOL colluvial, issu d’éboulis calcaires |
| **Soil name (WRB, 2015)** | | | Calcaric Cambisol (Colluvic) |
| **Humus form (Zanella et al., 2018)** | | | Oligomull |
| **Horizon sequence (Référentiel pédologique, 2009)** | | | OL / OF-Aca / Aca-Sca / Sca1 / Sca2 |
| **Site name** | | | BE4 |
|  | | | |
| **Station parameters** | | | |
| **Location** | Schwändiwald | 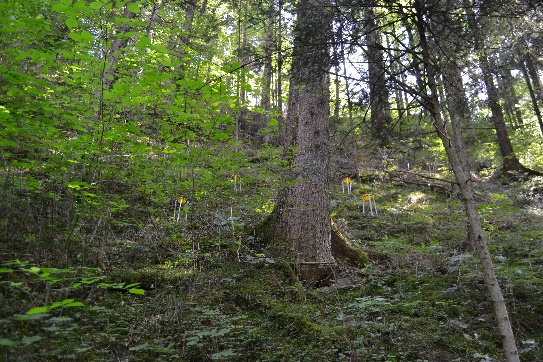 | |
| **Year of sampling** | 2018 |  |  |
| **Altitude** | 870 m |  |  |
| **Slope** | 35° |  |  |
| **Aspect** | 220° |  |  |
| **Topography** | Steep slope |  |  |
| **Geology** | Calcareous scree |  |  |
| **Vegetation type** | *Lonicero-Fagenion* |  |  |
|  | | | |
| **Soil profile** | | | |
| 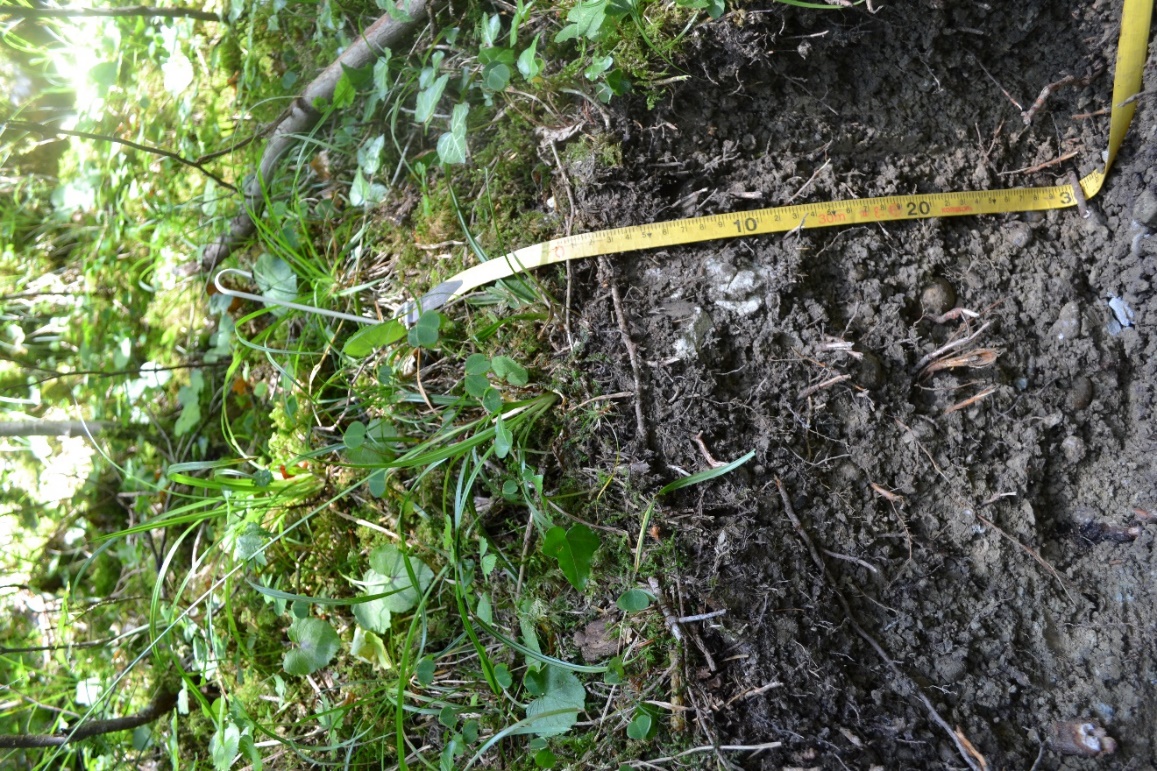 | | | |

| **Soil classification** | | | |
| --- | --- | --- | --- |
| **Soil name (Référentiel pédologique, 2009)** | | | CALCOSOL issu de molasse |
| **Soil name (WRB, 2015)** | | | Calcaric Cambisol |
| **Humus form (Zanella et al., 2018)** | | | Oligomull |
| **Horizon sequence (Référentiel pédologique, 2009)** | | | OL / BryoOL-OF-Aca / Aca / Aca-Sca-Cca |
| **Site name** | | | BE5 |
|  | | | |
| **Station parameters** | | | |
| **Location** | Zapfenmoos | 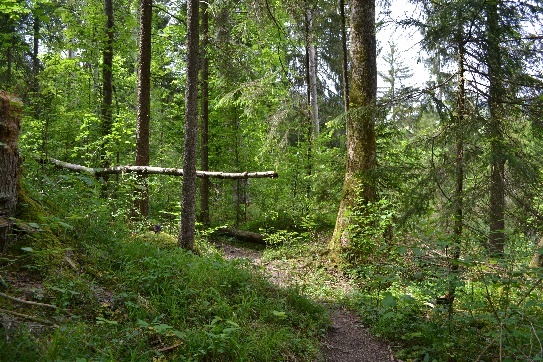 | |
| **Year of sampling** | 2018 |  |  |
| **Altitude** | 780 m |  |  |
| **Slope** | 15° |  |  |
| **Aspect** | 0° |  |  |
| **Topography** | Light slope |  |  |
| **Geology** | Molasse |  |  |
| **Vegetation type** | *Lonicero-Fagenion* |  |  |
|  | | | |
| **Soil profile** | | | |
| 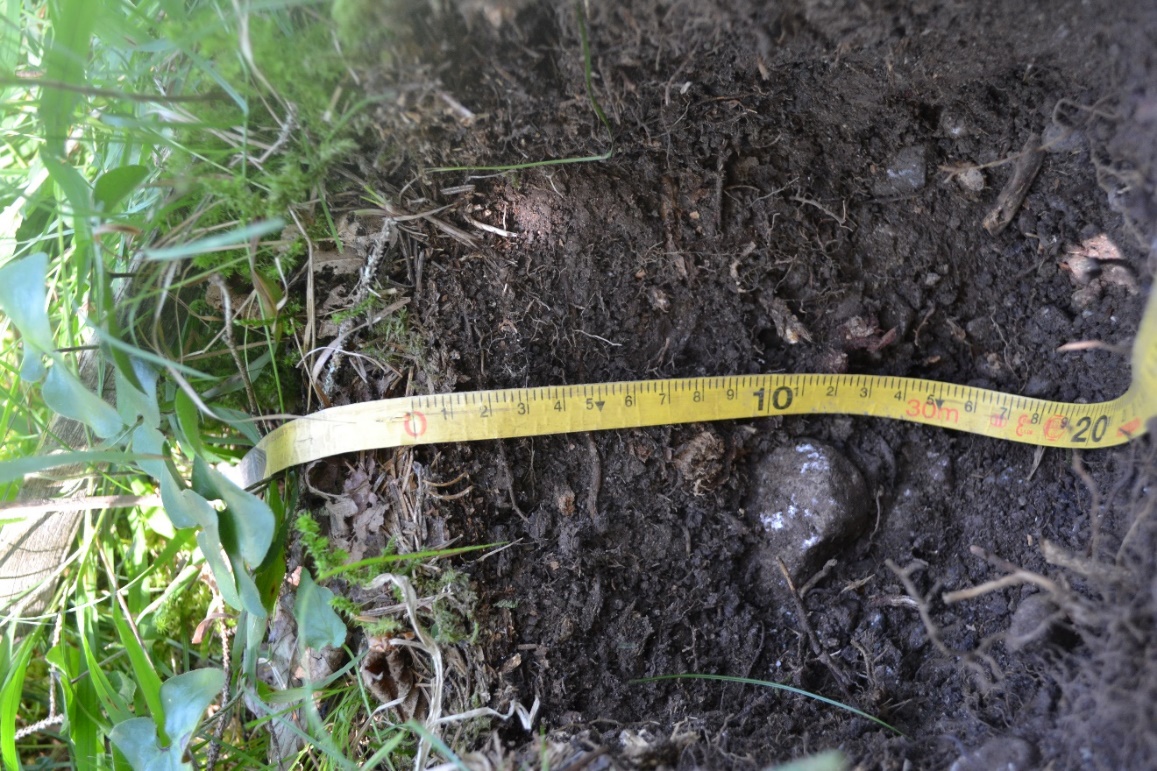 | | | |

| **Soil classification** | | | |
| --- | --- | --- | --- |
| **Soil name (Référentiel pédologique, 2009)** | | | CALCOSOL colluvial, issu d’éboulis calcaires |
| **Soil name (WRB, 2015)** | | | Calcaric Cambisol (Colluvic) |
| **Humus form (Zanella et al., 2018)** | | | Oligomull |
| **Horizon sequence (Référentiel pédologique, 2009)** | | | OL / OF-Aca / Aca-Sca / Cca |
| **Site name** | | | BE6 |
|  | | | |
| **Station parameters** | | | |
| **Location** | Sahlenweidli | 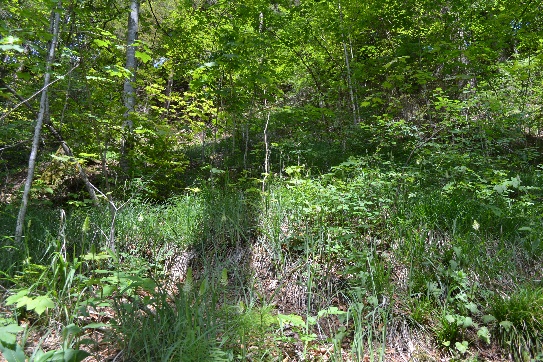 | |
| **Year of sampling** | 2018 |  |  |
| **Altitude** | 915 m |  |  |
| **Slope** | 40° |  |  |
| **Aspect** | 160° |  |  |
| **Topography** | Steep slope |  |  |
| **Geology** | Calcareous scree |  |  |
| **Vegetation type** | *Lonicero-Fagenion* |  |  |
|  | | | |
| **Soil profile** | | | |
| 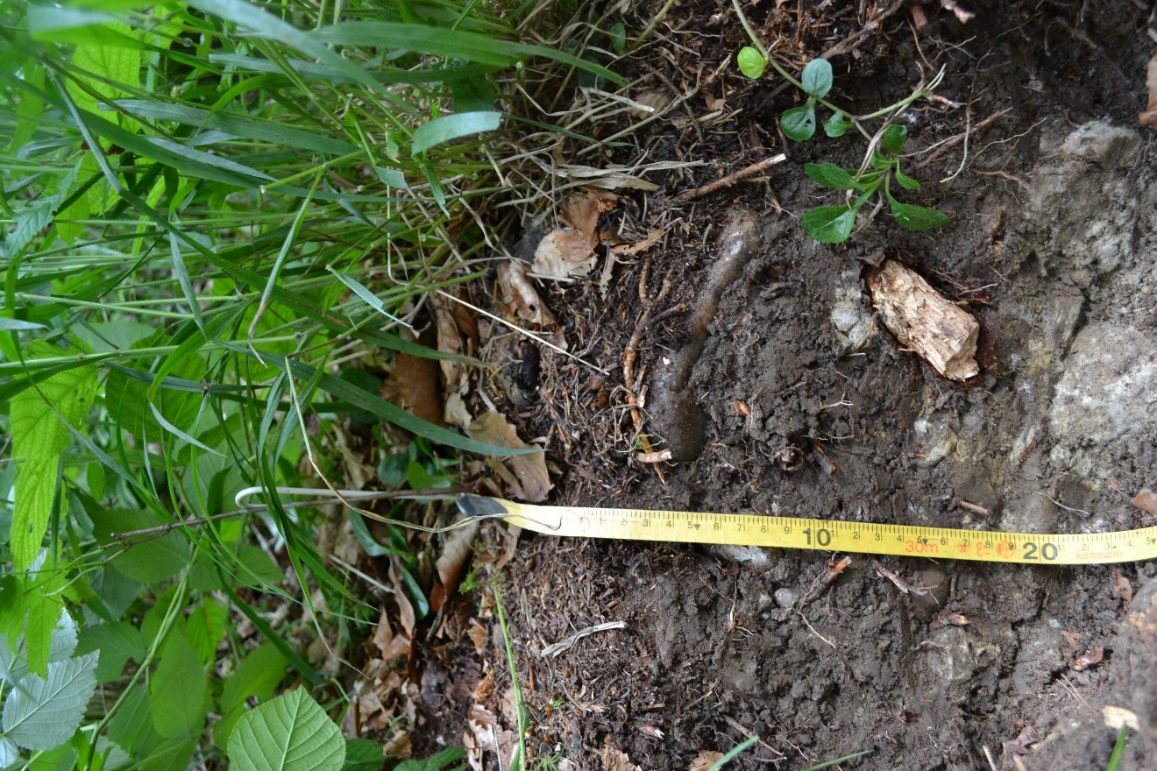 | | | |

| **Soil classification** | | | |
| --- | --- | --- | --- |
| **Soil name (Référentiel pédologique, 2009)** | | | CALCOSOL à horizon A hémiorganique, colluvial, issu d’éboulis calcaires |
| **Soil name (WRB, 2015)** | | | Calcaric Cambisol (Colluvic, Humic) |
| **Humus form (Zanella et al., 2018)** | | | Mesomull |
| **Horizon sequence (Référentiel pédologique, 2009)** | | | OL / Acaho / Acaho-Sca-Cca / Sca-Cca |
| **Site name** | | | BE7 |
|  | | | |
| **Station parameters** | | | |
| **Location** | Gasterntal | 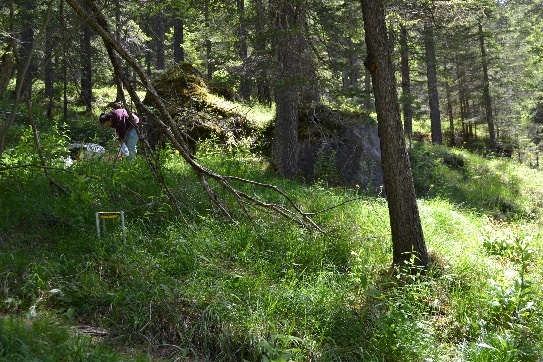 | |
| **Year of sampling** | 2018 |  |  |
| **Altitude** | 1540 m |  |  |
| **Slope** | 17° |  |  |
| **Aspect** | 230° |  |  |
| **Topography** | Slope with flat areas |  |  |
| **Geology** | Calcareous scree |  |  |
| **Vegetation type** | *Vaccinio-Piceion* |  |  |
|  | | | |
| **Soil profile** | | | |
| 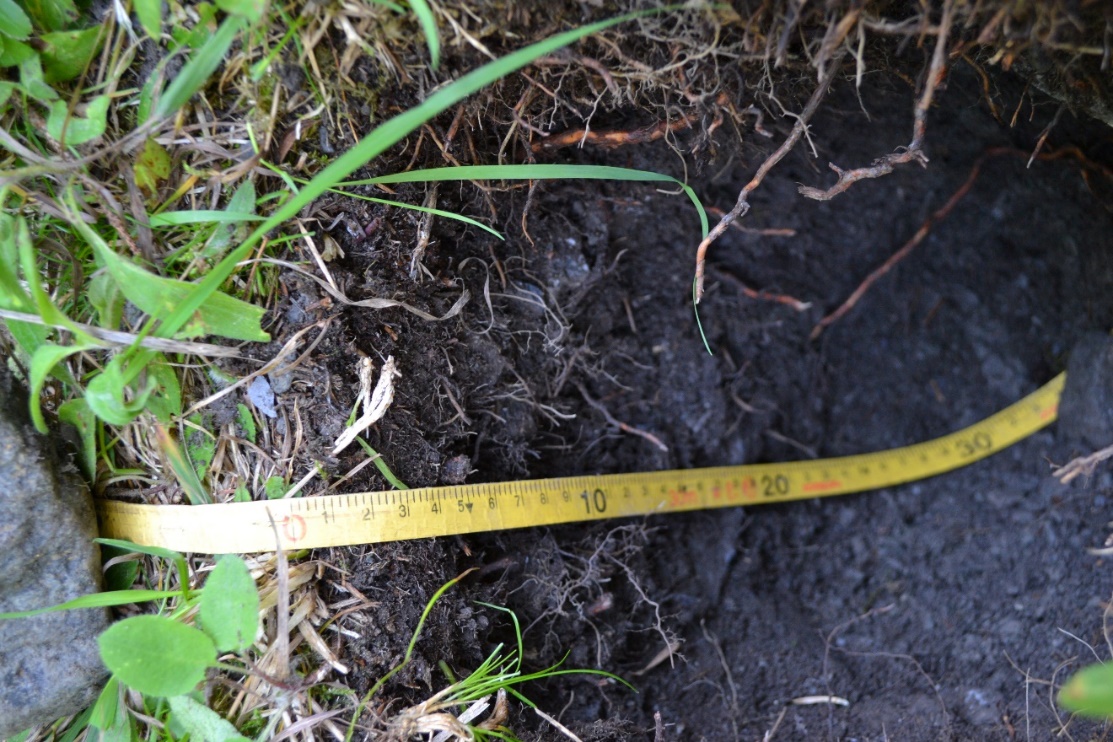 | | | |

| **Soil classification** | | | |
| --- | --- | --- | --- |
| **Soil name (Référentiel pédologique, 2009)** | | | RENDOSOL colluvial, issu d’éboulis calcaires |
| **Soil name (WRB, 2015)** | | | Rendzic Calcaric Leptosol (Colluvic) |
| **Humus form (Zanella et al., 2018)** | | | Hemimoder |
| **Horizon sequence (Référentiel pédologique, 2009)** | | | vOL-nOL-BryoOL / OF-[OH] / Acaho-Cca |
| **Site name** | | | BL1 |
|  | | | |
| **Station parameters** | | | |
| **Location** | Langenbruck | 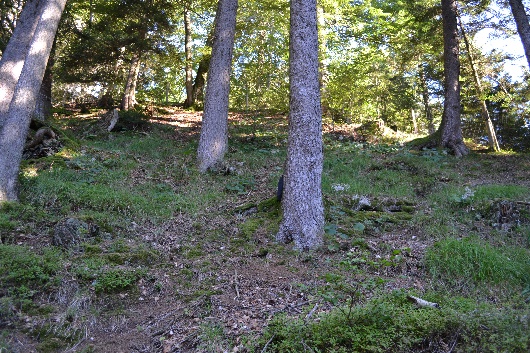 | |
| **Year of sampling** | 2016 and 2021 |  |  |
| **Altitude** | 933 m |  |  |
| **Slope** | 35° |  |  |
| **Aspect** | 156° |  |  |
| **Topography** | Steep slope |  |  |
| **Geology** | Calcareous scree |  |  |
| **Vegetation type** | *Abieti-Fagenion* |  |  |
|  | | | |
| **Soil profile** | | | |
| 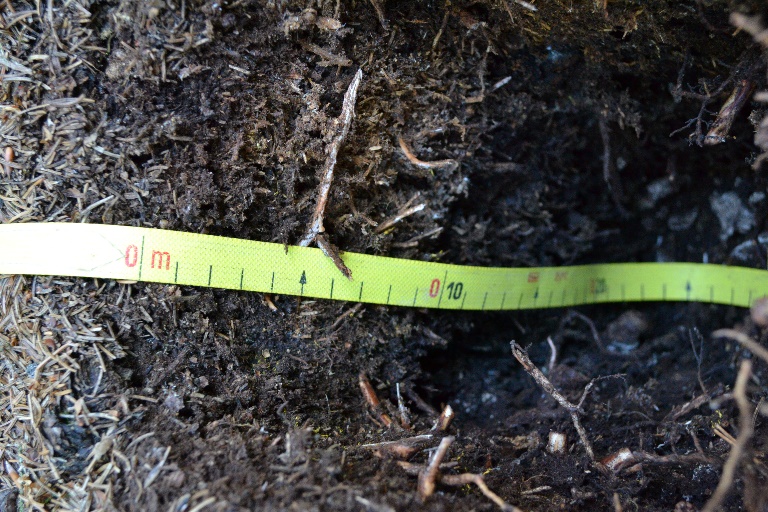 | | | |

| **Soil classification** | | | |
| --- | --- | --- | --- |
| **Soil name (Référentiel pédologique, 2009)** | | | DOLOMITOSOL |
| **Soil name (WRB, 2015)** | | | Dolomitic Cambisol |
| **Humus form (Zanella et al., 2018)** | | | Oligomull |
| **Horizon sequence (Référentiel pédologique, 2009)** | | | nOL-vOL / Ado1 / Ado2 / Sdo |
| **Site name** | | | GR1 |
|  | | | |
| **Station parameters** | | | |
| **Location** | Schmitten | 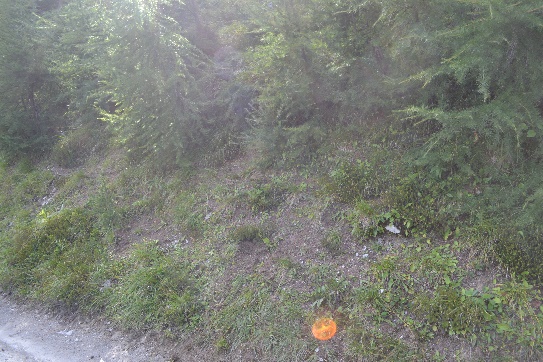 | |
| **Year of sampling** | 2019 and 2021 |  |  |
| **Altitude** | 1927 m |  |  |
| **Slope** | 10° |  |  |
| **Aspect** | 88° |  |  |
| **Topography** | Light slope, edge of a forest road |  |  |
| **Geology** | Dolomite |  |  |
| **Vegetation type** | *Erico-Pinion unicinatae* |  |  |
|  | | | |
| **Soil profile** | | | |
| 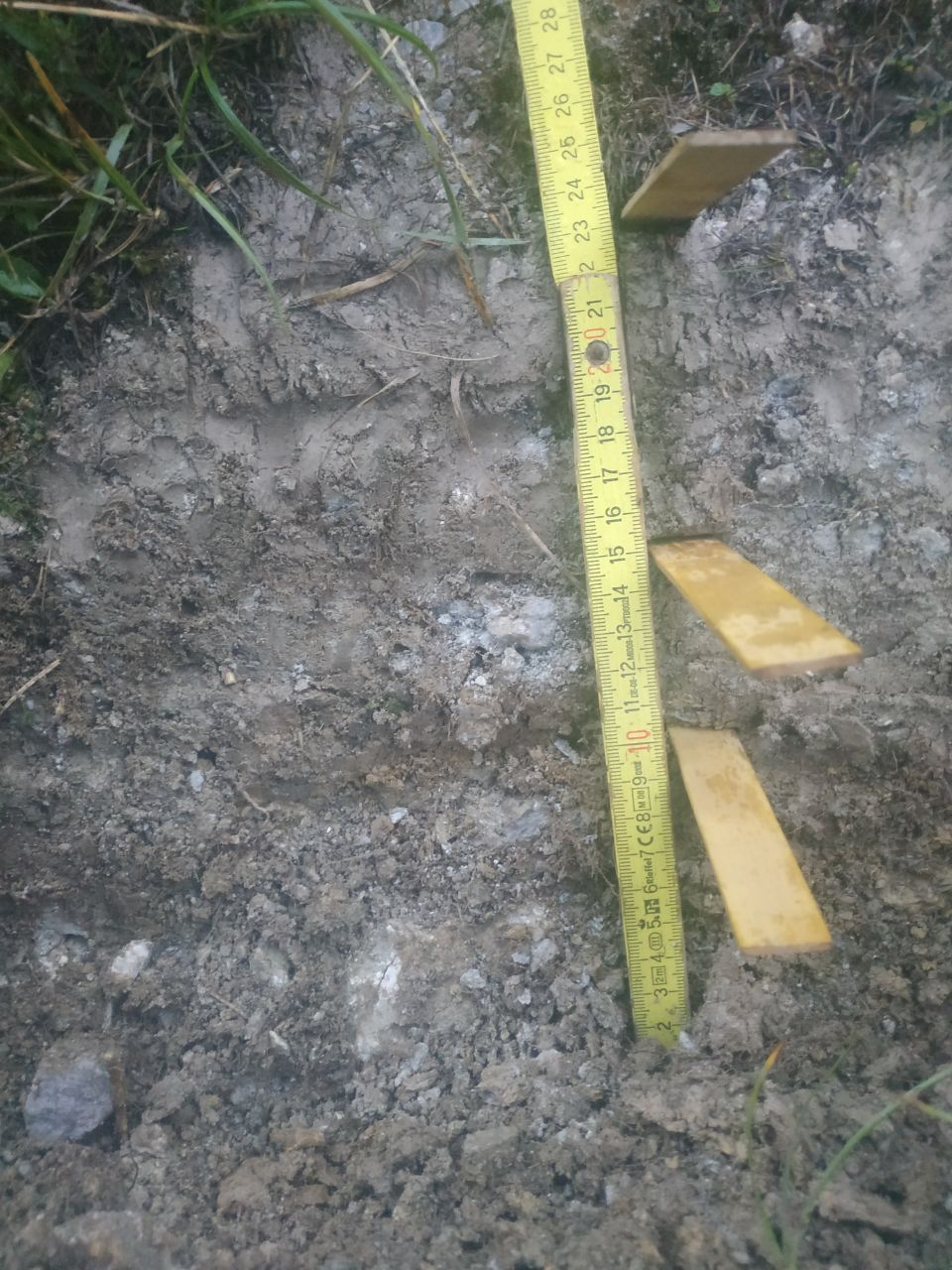 | | | |

| **Soil classification** | | |
| --- | --- | --- |
| **Soil name (Référentiel pédologique, 2009)** | | DOLOMITOSOL à horizons gypsiques |
| **Soil name (WRB, 2015)** | | Gypsiric Dolomitic Cambisol |
| **Humus form (Zanella et al., 2018)** | | Dysmull |
| **Horizon sequence (Référentiel pédologique, 2009)** | | [nOL]-[vOL]-OF / Adoy / Cdo1 / Cdo2 / Cdo3 |
| **Site name** | | GR2 |
|  | | |
| 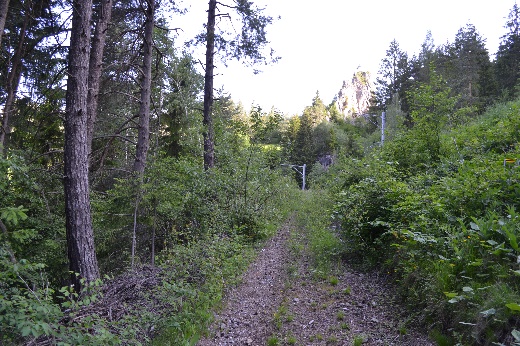**Station parameters** | | |
| **Location** | Filisur (Bim Schloss) |  |
| **Year of sampling** | 2019 |  |
| **Altitude** | 1128 m |  |
| **Slope** | 32° |  |
| **Aspect** | 253° |  |
| **Topography** | Steep slope between a forest road and railroad |  |
| **Geology** | Dolomite |  |
| **Vegetation type** | *Cephalanthero-Fagenion* |  |
|  | | |
| **Soil profile** | | |
| 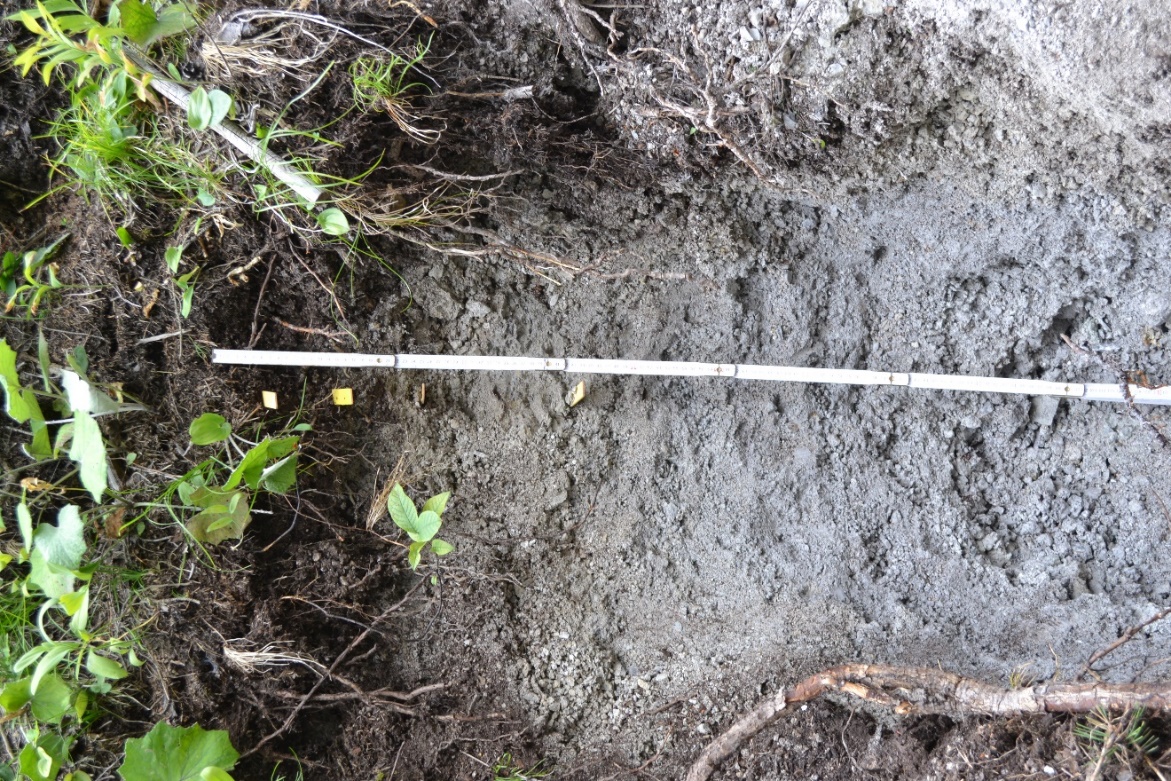 | | |

| **Soil classification** | | | |
| --- | --- | --- | --- |
| **Soil name (Référentiel pédologique, 2009)** | | | DOLOMITOSOL à horizons gypsiques |
| **Soil name (WRB, 2015)** | | | Gypsiric Dolomitic Cambisol |
| **Humus form (Zanella et al., 2018)** | | | Eumull |
| **Horizon sequence (Référentiel pédologique, 2009)** | | | nOL / Adoy / Sdoy |
| **Site name** | | | GR3 |
|  | | | |
| **Station parameters** | | | |
| **Location** | Lenzerheide | 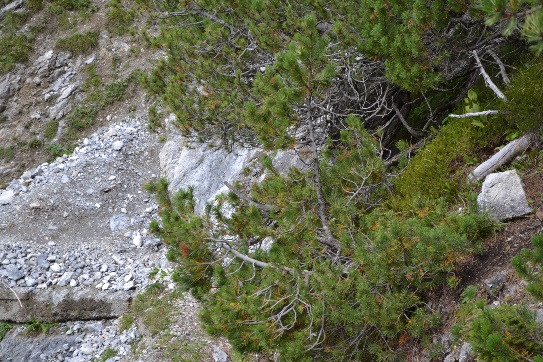 | |
| **Year of sampling** | 2019 and 2021 |  |  |
| **Altitude** | 1922 m |  |  |
| **Slope** | 35° |  |  |
| **Aspect** | 218° |  |  |
| **Topography** | Steep slope, scree |  |  |
| **Geology** | Dolomite |  |  |
| **Vegetation type** | *Erico-Pinion sylvestris* |  |  |
|  | | | |
| **Soil profile** | | | |
| 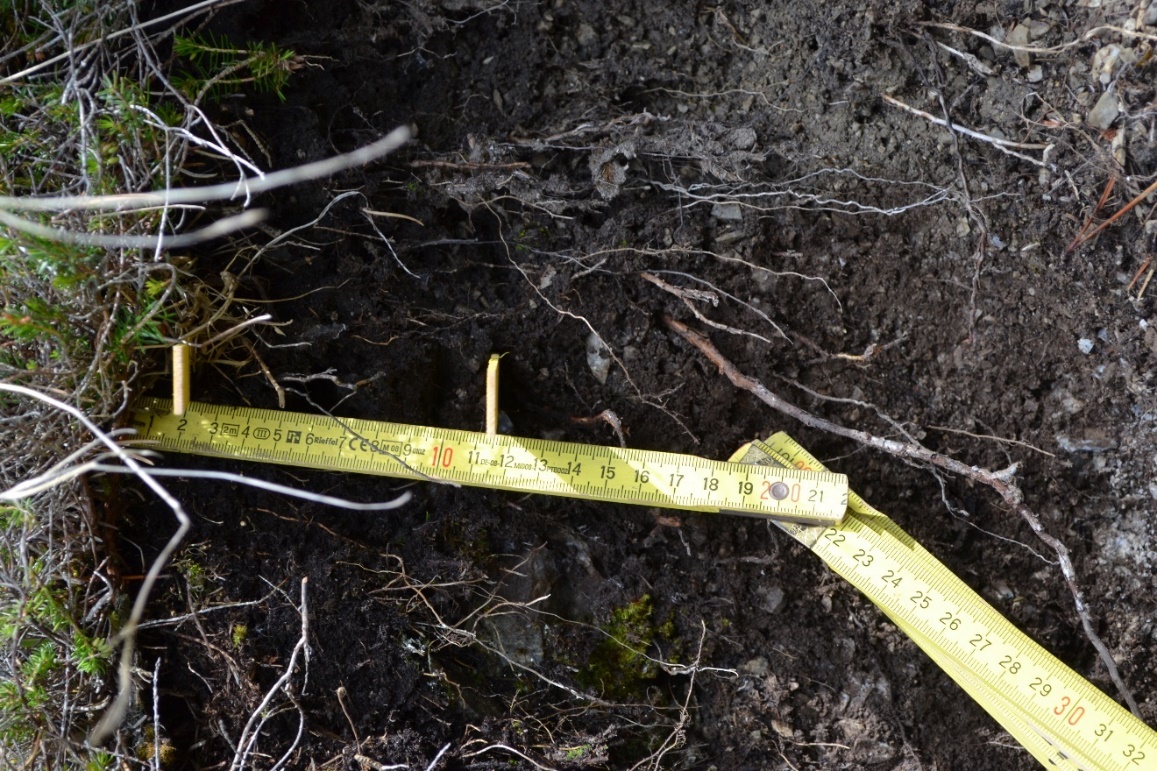 | | | |

| **Soil classification** | | | |
| --- | --- | --- | --- |
| **Soil name (Référentiel pédologique, 2009)** | | | CALCOSOL à horizon A hémiorganique |
| **Soil name (WRB, 2015)** | | | Calcaric Cambisol (Humic) |
| **Humus form (Zanella et al., 2018)** | | | Pachyamphi |
| **Horizon sequence (Référentiel pédologique, 2009)** | | | vOL-nOL / BryoOL-OF-OH / Acaho / Sca |
| **Site name** | | | GR4 |
|  | | | |
| **Station parameters** | | | |
| **Location** | Bonaduz | 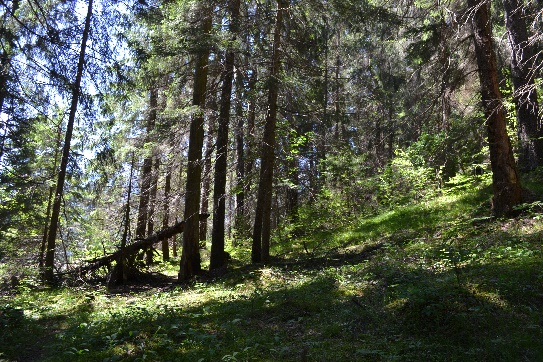 | |
| **Year of sampling** | 2019 |  |  |
| **Altitude** | 880 m |  |  |
| **Slope** | 30° |  |  |
| **Aspect** | 94° |  |  |
| **Topography** | Slopes and flat areas |  |  |
| **Geology** | Mixed moraine |  |  |
| **Vegetation type** | *Cephalanthero-Fagenion* |  |  |
|  | | | |
| **Soil profile** | | | |
| 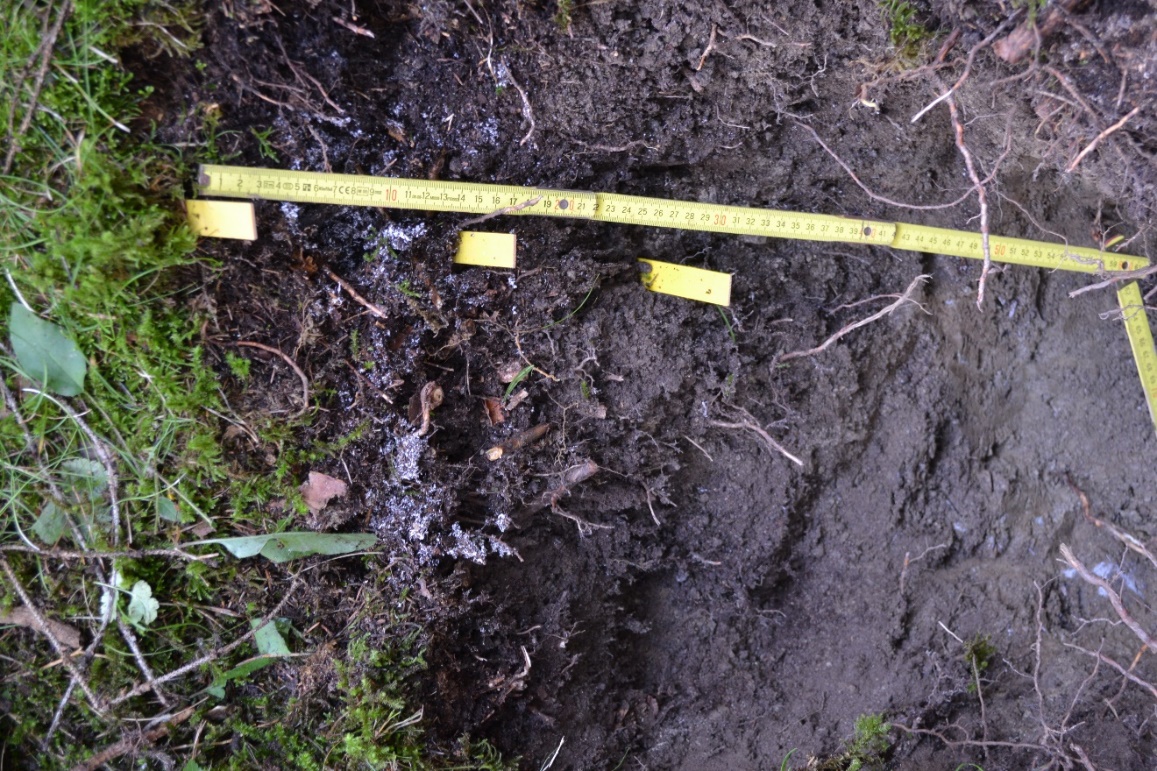 | | | |

| **Soil classification** | | | |
| --- | --- | --- | --- |
| **Soil name (Référentiel pédologique, 2009)** | | | DOLOMITOSOL à horizons gypsiques |
| **Soil name (WRB, 2015)** | | | Gypsiric Dolomitic Cambisol |
| **Humus form (Zanella et al., 2018)** | | | Rhizoamphi |
| **Horizon sequence (Référentiel pédologique, 2009)** | | | nOL-[vOL] / OF-[OH] / Adoy / Adoy-Sdoy |
| **Site name** | | | GR5 |
|  | | | |
| **Station parameters** | | | |
| **Location** | Bergün | 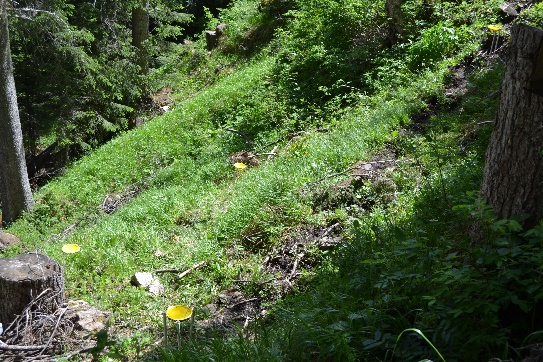 | |
| **Year of sampling** | 2019 |  |  |
| **Altitude** | 1700 m |  |  |
| **Slope** | 38° |  |  |
| **Aspect** | 350° |  |  |
| **Topography** | Steep slope and edge of a road |  |  |
| **Geology** | NA |  |  |
| **Vegetation type** | *Vaccinio-Piceion* |  |  |
|  | | | |
| **Soil profile** | | | |
| 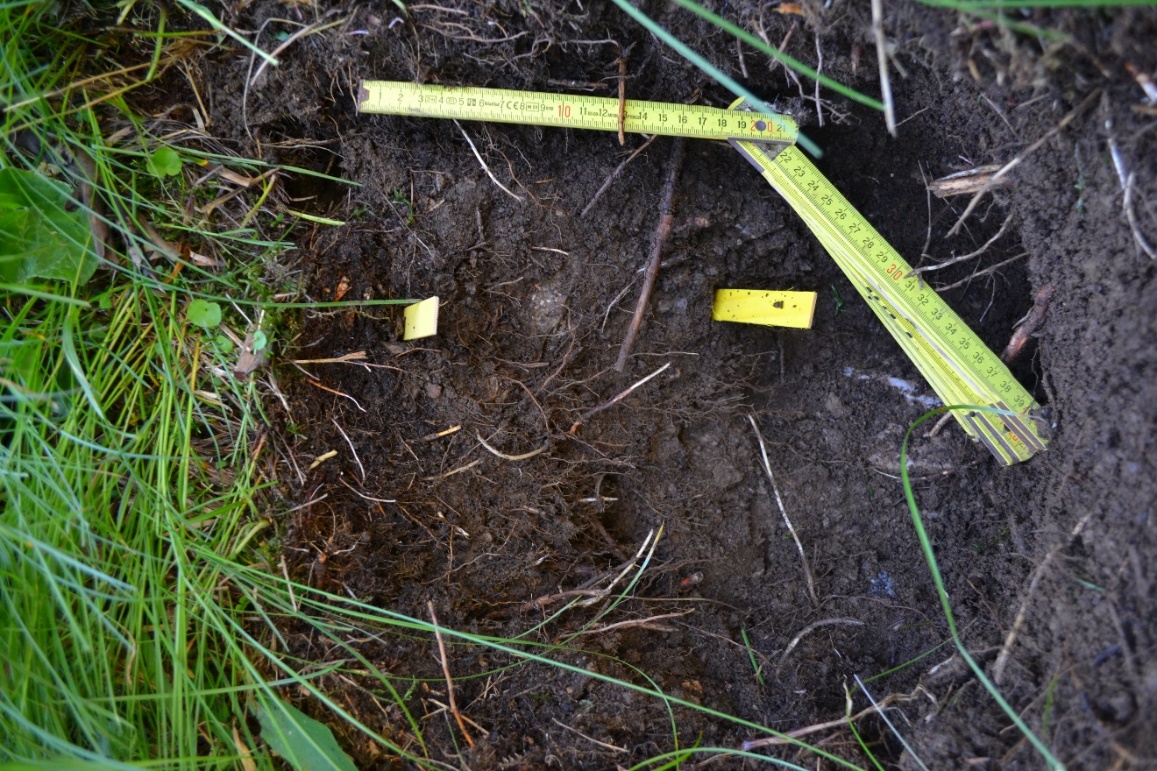 | | | |

| **Soil classification** | | | |
| --- | --- | --- | --- |
| **Soil name (Référentiel pédologique, 2009)** | | | DOLOMITOSOL |
| **Soil name (WRB, 2015)** | | | Dolomitic Cambisol |
| **Humus form (Zanella et al., 2018)** | | | Mesomull |
| **Horizon sequence (Référentiel pédologique, 2009)** | | | OF / Ado / Ado-Sdo / Sdo |
| **Site name** | | | GR6 |
|  | | | |
| **Station parameters** | | | |
| **Location** | Zernez | 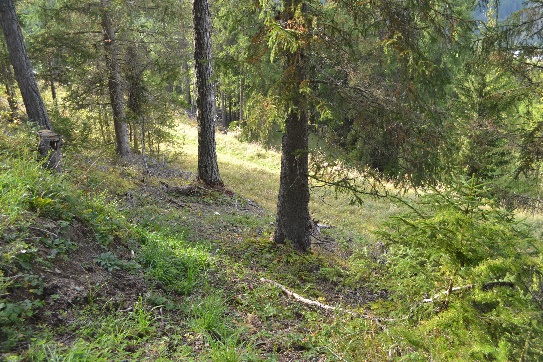 | |
| **Year of sampling** | 2019 and 2021 |  |  |
| **Altitude** | 1526 m |  |  |
| **Slope** | 15° |  |  |
| **Aspect** | 340° |  |  |
| **Topography** | Light slope, edge of a forest road |  |  |
| **Geology** | NA |  |  |
| **Vegetation type** | *Vaccinio-Piceion* |  |  |
|  | | | |
| **Soil profile** | | | |
| 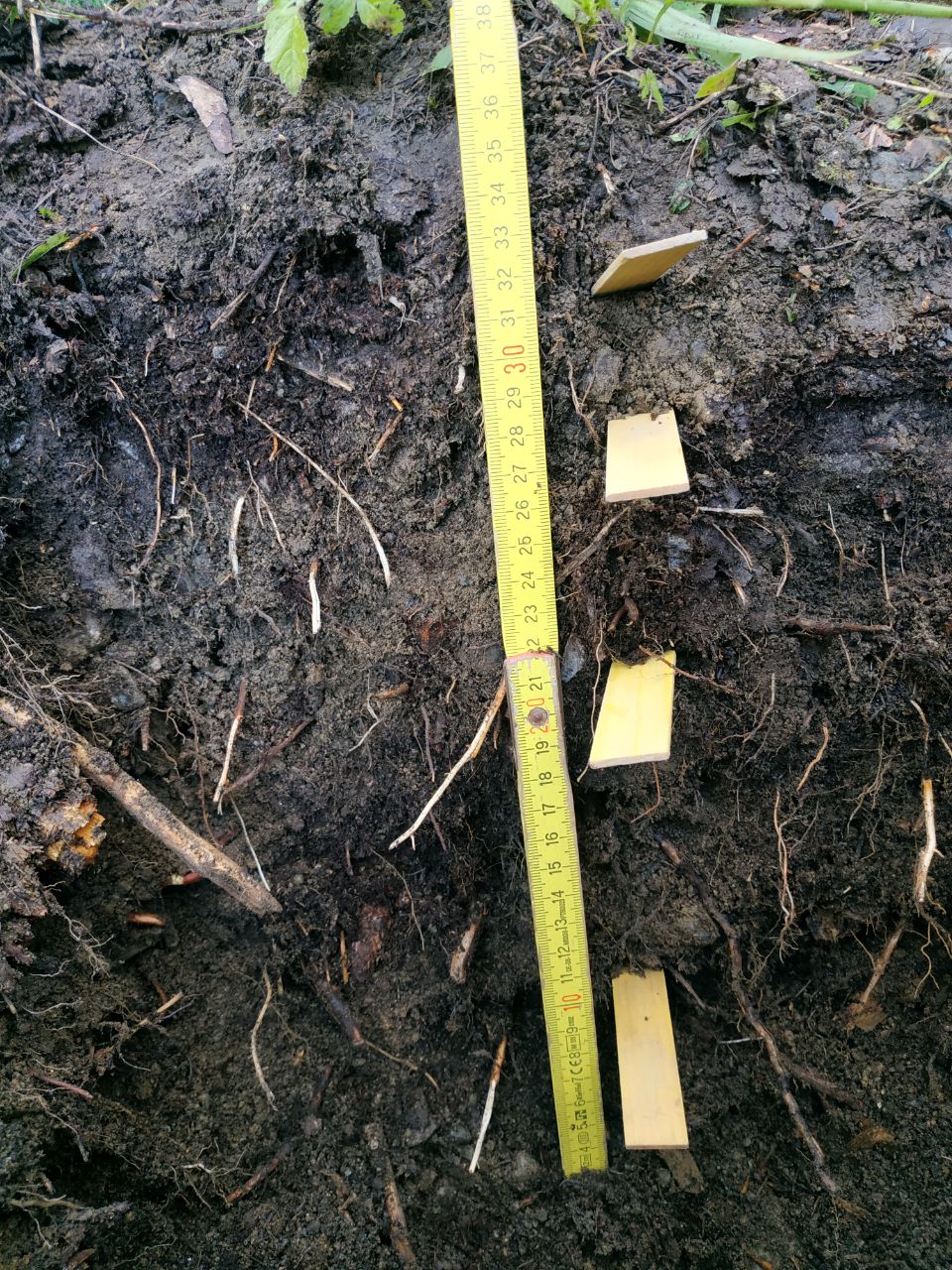 | | | |

| **Soil classification** | | | |
| --- | --- | --- | --- |
| **Soil name (Référentiel pédologique, 2009)** | | | CALCOSOL hypocalcaire, colluvial, issu d’éboulis calcaires |
| **Soil name (WRB, 2015)** | | | Calcaric Cambisol (Colluvic) |
| **Humus form (Zanella et al., 2018)** | | | Oligomull |
| **Horizon sequence (Référentiel pédologique, 2009)** | | | [nOL]-[vOL] / S*-OF / [OF]-Aca / Aca-Sca / Dca |
| **Site name** | | | GR7 |
|  | | | |
| **Station parameters** | | | |
| **Location** | Ruina Belfort | 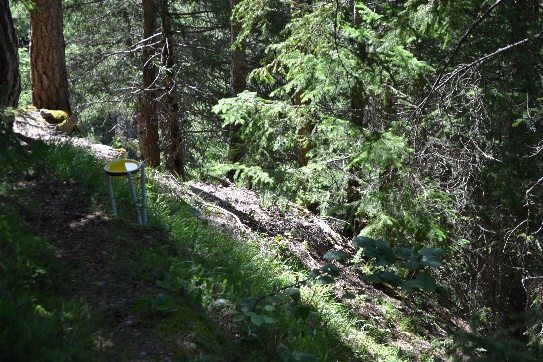 | |
| **Year of sampling** | 2019 and 2021 |  |  |
| **Altitude** | 1153 m |  |  |
| **Slope** | 35° |  |  |
| **Aspect** | 205° |  |  |
| **Topography** | Very steep slope |  |  |
| **Geology** | Calcareous scree |  |  |
| **Vegetation type** | *Erico-Pinion sylvestris* |  |  |
|  | | | |
| **Soil profile** | | | |
| 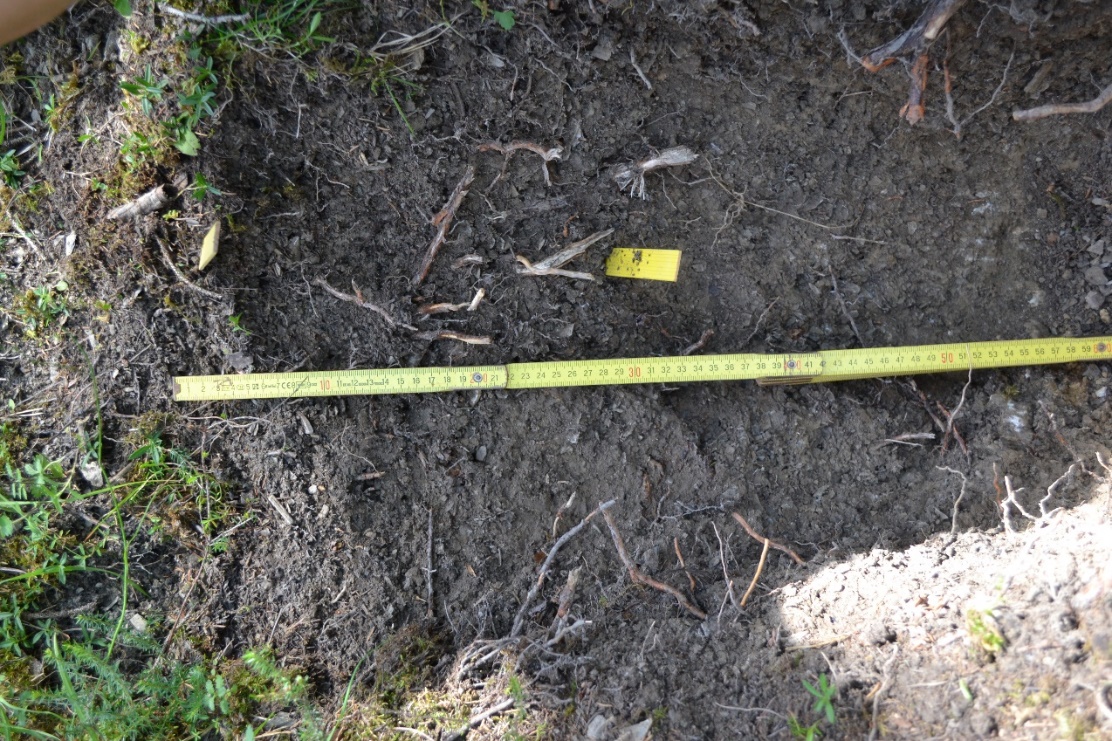 | | | |

| **Soil classification** | | | |
| --- | --- | --- | --- |
| **Soil name (Référentiel pédologique, 2009)** | | | CALCOSOL colluvial, leptique, issu d’éboulis calcaires |
| **Soil name (WRB, 2015)** | | | Calcaric Cambisol (Colluvic) |
| **Humus form (Zanella et al., 2018)** | | | Dysmoder |
| **Horizon sequence (Référentiel pédologique, 2009)** | | | [BryoOL]-[vOL]-[nOL] / OF-OH / Aca / Cca-[Sca] |
| **Site name** | | | NE1 |
|  | | | |
| **Station parameters** | | | |
| **Location** | Creux-du-Van | 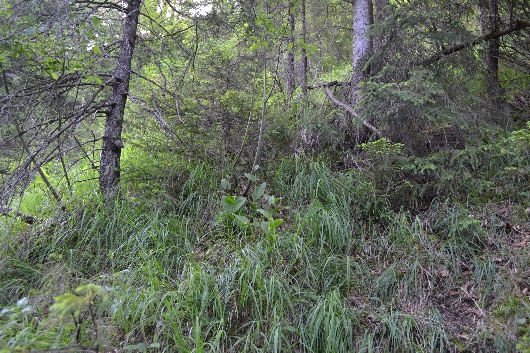 | |
| **Year of sampling** | 2016 and 2018 |  |  |
| **Altitude** | 1166 m |  |  |
| **Slope** | 45° |  |  |
| **Aspect** | 295° |  |  |
| **Topography** | Steep slope |  |  |
| **Geology** | Calcareous scree |  |  |
| **Vegetation type** | *Lonicero-Fagenion* |  |  |
|  | | | |
| **Soil profile** | | | |
| 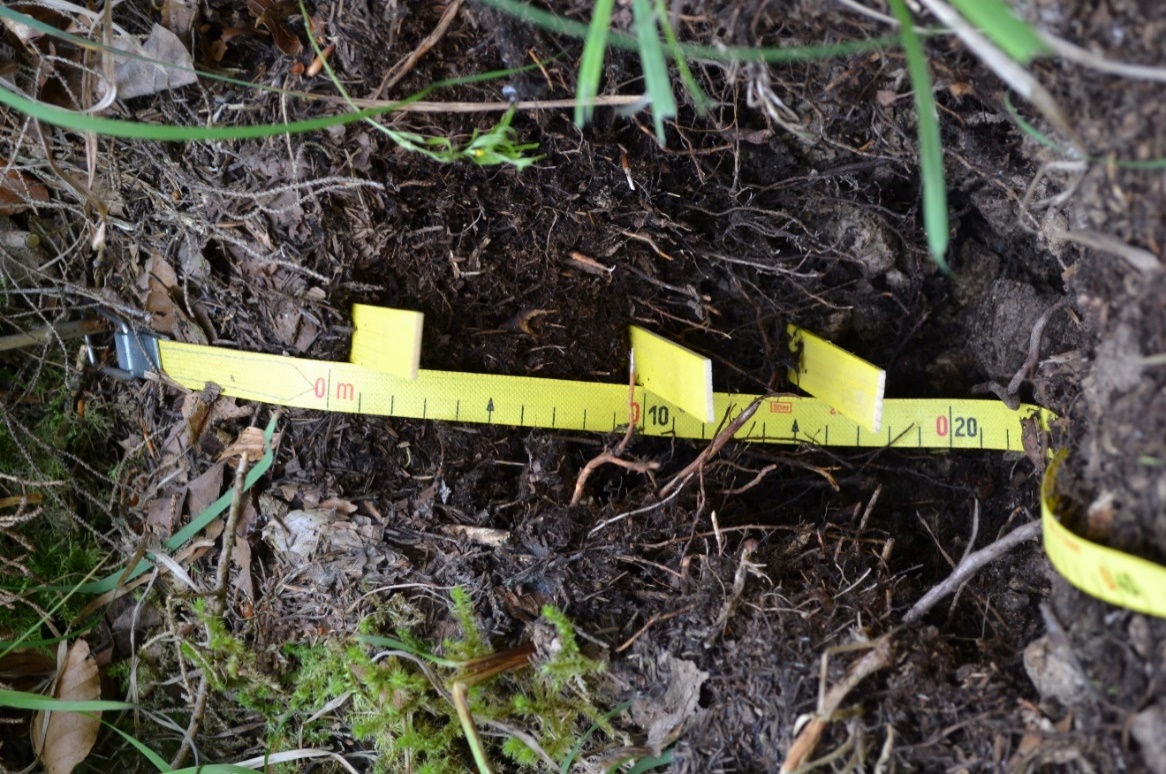 | | | |

| **Soil classification** | | | |
| --- | --- | --- | --- |
| **Soil name (Référentiel pédologique, 2009)** | | | RENDOSOL colluvial, issu d’éboulis calcaires |
| **Soil name (WRB, 2015)** | | | Rendzic Calcaric Leptosol (Colluvic) |
| **Humus form (Zanella et al., 2018)** | | | Dysmoder |
| **Horizon sequence (Référentiel pédologique, 2009)** | | | vOL-BryOL / OF-OH / Aca / Dca-[Aca] |
| **Site name** | | | NE2 |
|  | | | |
| **Station parameters** | | | |
| **Location** | Creux-du-Van | 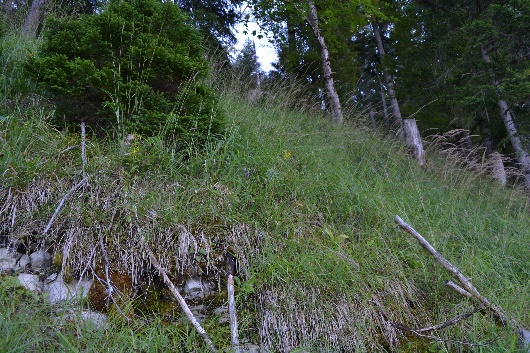 | |
| **Year of sampling** | 2016 and 2021 |  |  |
| **Altitude** | 1187 m |  |  |
| **Slope** | 55° |  |  |
| **Aspect** | 336° |  |  |
| **Topography** | Steep slope with a rocky break |  |  |
| **Geology** | Calcareous scree |  |  |
| **Vegetation type** | *Abieti-Fagenion* |  |  |
|  | | | |
| **Soil profile** | | | |
| 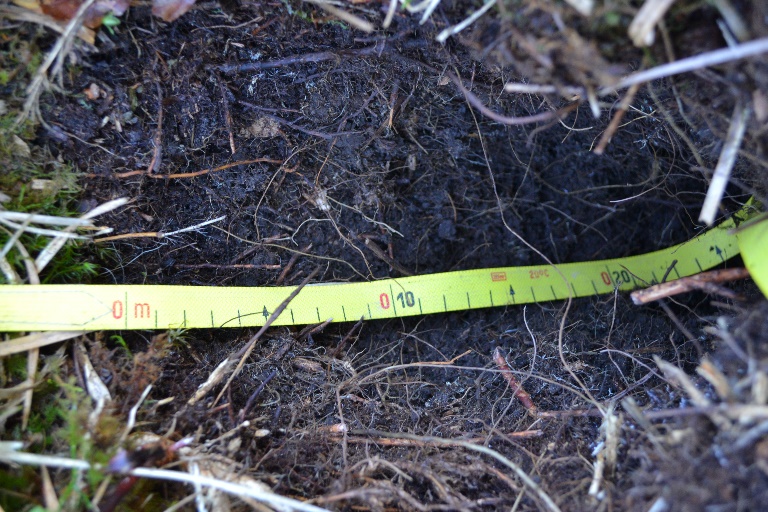 | | | |

| **Soil classification** | | | |
| --- | --- | --- | --- |
| **Soil name (Référentiel pédologique, 2009)** | | | DOLOMITOSOL |
| **Soil name (WRB, 2015)** | | | Dolomitic Cambisol |
| **Humus form (Zanella et al., 2018)** | | | Oligomull |
| **Horizon sequence (Référentiel pédologique, 2009)** | | | nOL / OF / Ado |
| **Site name** | | | TI1 |
|  | | | |
| **Station parameters** | | | |
| **Location** | Airolo | 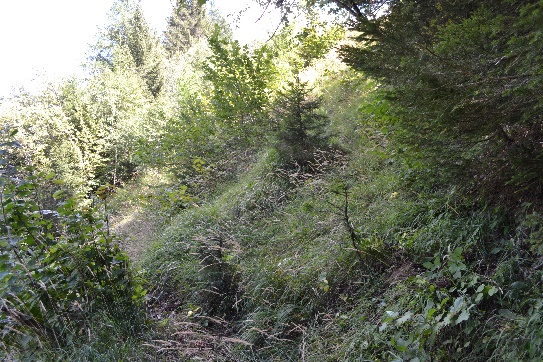 | |
| **Year of sampling** | 2019 and 2021 |  |  |
| **Altitude** | 1276 m |  |  |
| **Slope** | 20° |  |  |
| **Aspect** | 326° |  |  |
| **Topography** | North-facing slope |  |  |
| **Geology** | NA |  |  |
| **Vegetation type** | *Sambuco-Salicion* |  |  |
|  | | | |
| **Soil profile** | | | |
| 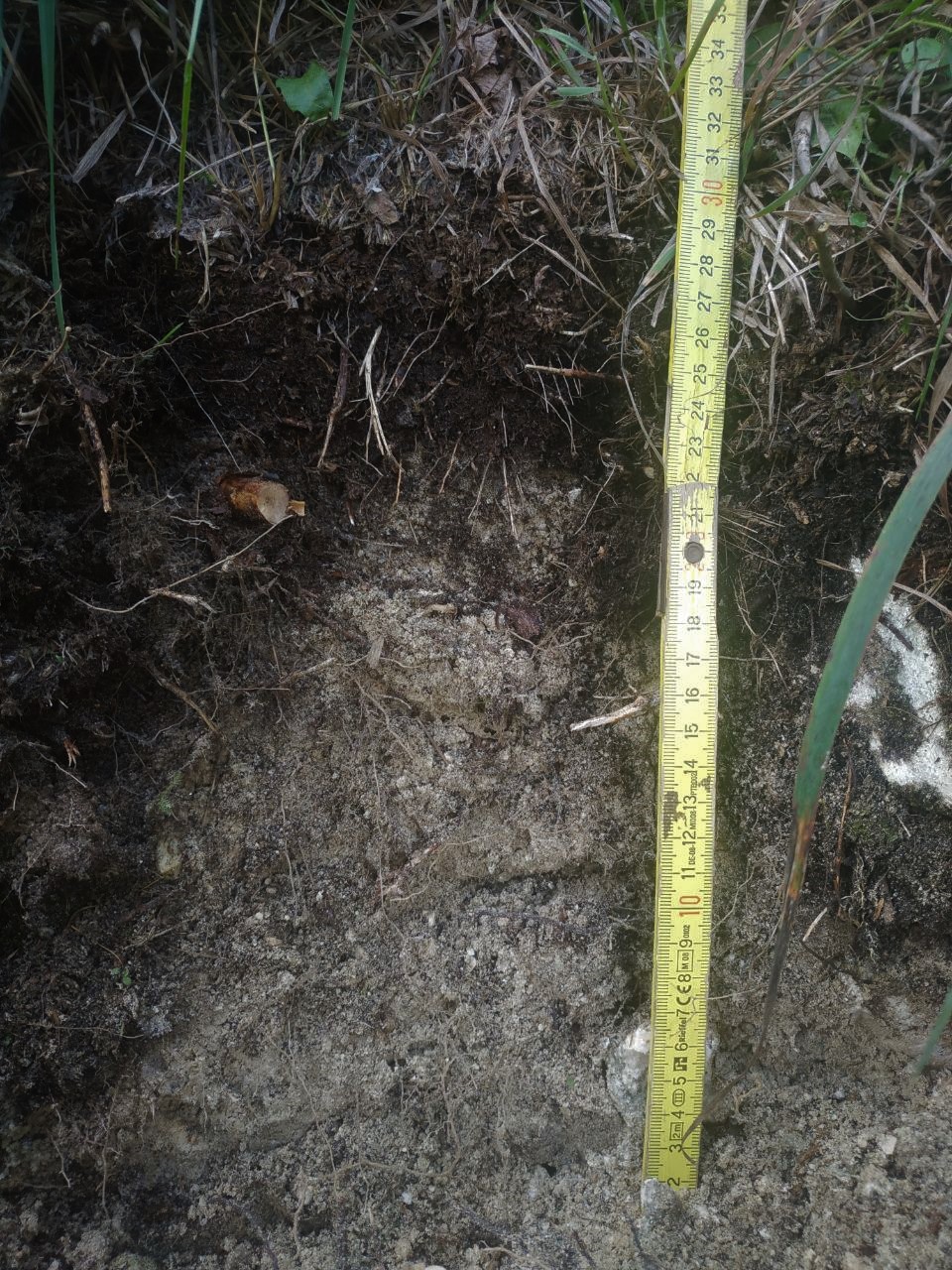 | | | |

| **Soil classification** | | | |
| --- | --- | --- | --- |
| **Soil name (Référentiel pédologique, 2009)** | | | DOLOMITOSOL |
| **Soil name (WRB, 2015)** | | | Dolomitic Cambisol |
| **Humus form (Zanella et al., 2018)** | | | Mesomull |
| **Horizon sequence (Référentiel pédologique, 2009)** | | | vOL / Ado / Ado-Sdo / Sdo1 / Sdo2 |
| **Site name** | | | TI2 |
|  | | | |
| **Station parameters** | | | |
| **Location** | Bedretto | 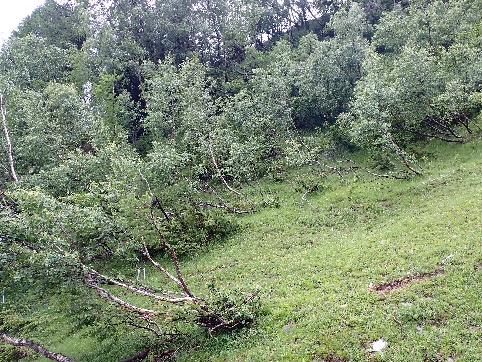 | |
| **Year of sampling** | 2019 and 2021 |  |  |
| **Altitude** | 1495 m |  |  |
| **Slope** | 45° |  |  |
| **Aspect** | 80° |  |  |
| **Topography** | Landslide cone |  |  |
| **Geology** | Dolomite |  |  |
| **Vegetation type** | *Vaccinio-Piceion* |  |  |
|  | | | |
| **Soil profile** | | | |
| 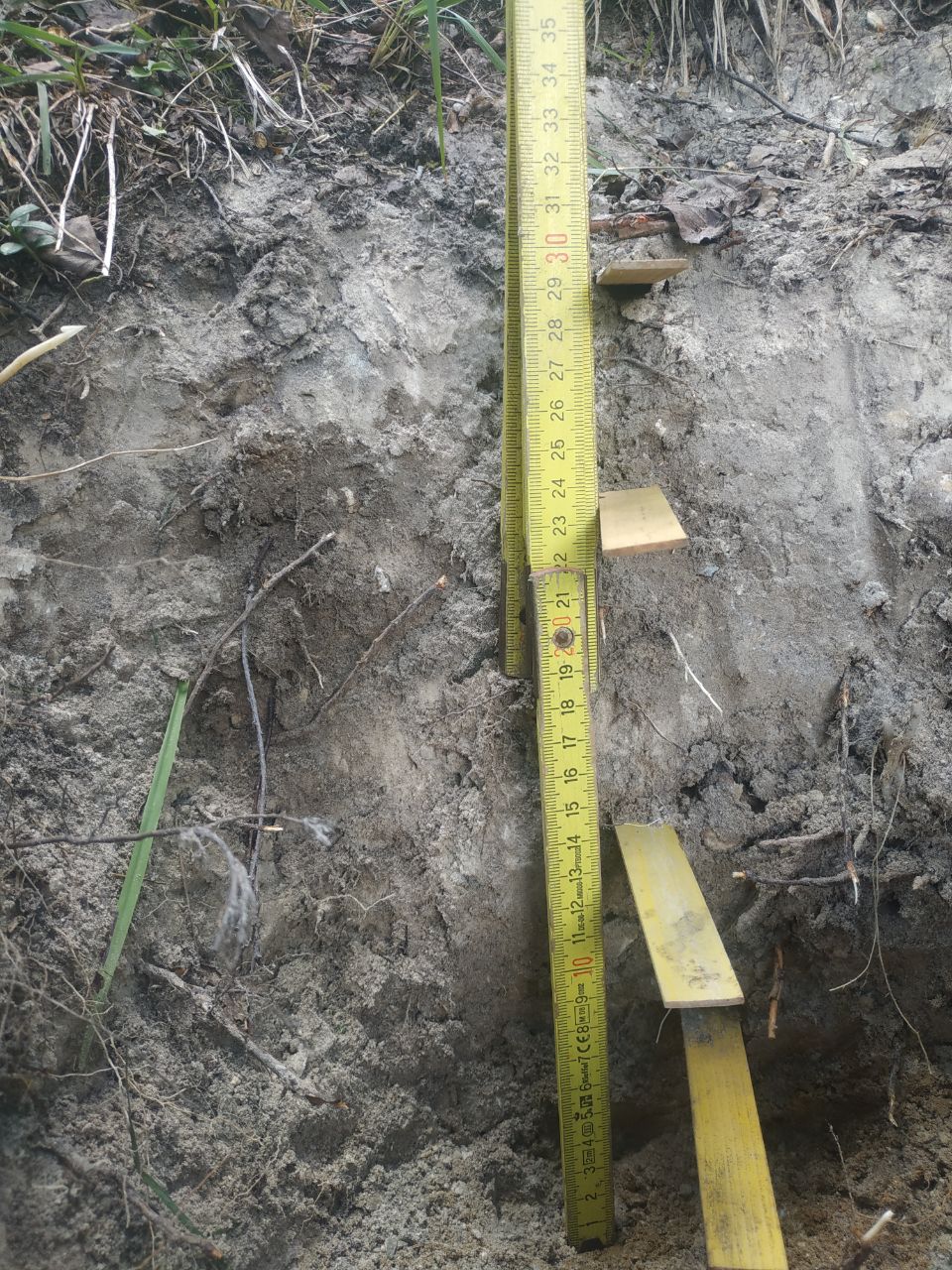 | | | |

| **Soil classification** | | | |
| --- | --- | --- | --- |
| **Soil name (Référentiel pédologique, 2009)** | | | CALCOSOL colluvial, issu d’éboulis calcaires |
| **Soil name (WRB, 2015)** | | | Calcaric Cambisol (Colluvic) |
| **Humus form (Zanella et al., 2018)** | | | Oligomull |
| **Horizon sequence (Référentiel pédologique, 2009)** | | | OL / OF-Aca / Aca-OF / Sca |
| **Site name** | | | VD1 |
|  | | | |
| **Station parameters** | | | |
| **Location** | Moulin d’Assens | 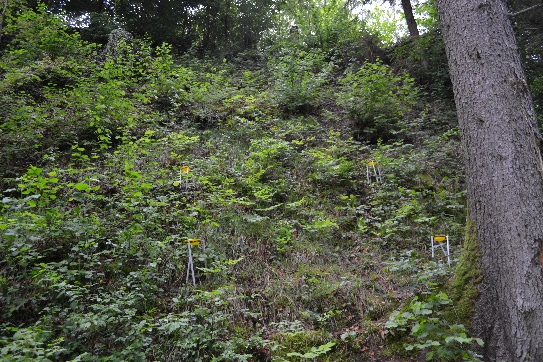 | |
| **Year of sampling** | 2018 |  |  |
| **Altitude** | 658 m |  |  |
| **Slope** | 40° |  |  |
| **Aspect** | 290° |  |  |
| **Topography** | Steep slope above a forest path |  |  |
| **Geology** | Calcareous scree |  |  |
| **Vegetation type** | *Galio-Fagenion* |  |  |
|  | | | |
| **Soil profile** | | | |
| 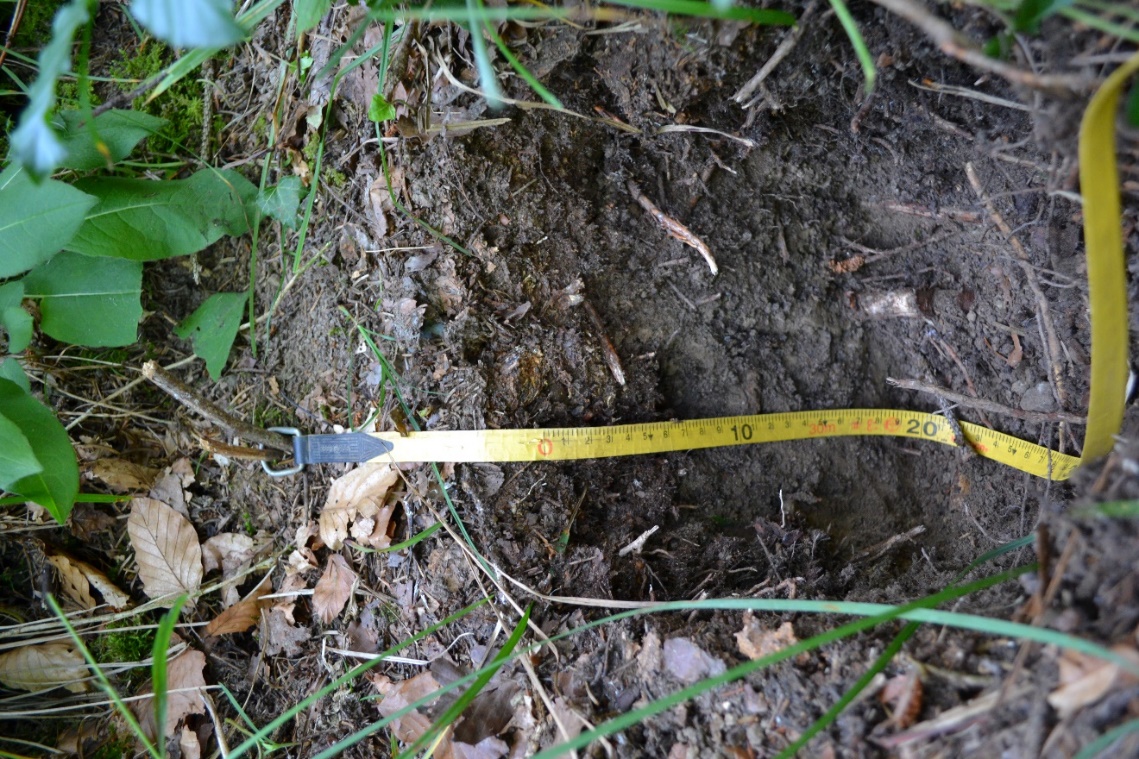 | | | |

| **Soil classification** | | | |
| --- | --- | --- | --- |
| **Soil name (Référentiel pédologique, 2009)** | | | RENDOSOL issu de molasse |
| **Soil name (WRB, 2015)** | | | Calcaric Cambisol (Humic) |
| **Humus form (Zanella et al., 2018)** | | | Mesomull |
| **Horizon sequence (Référentiel pédologique, 2009)** | | | OL / Acaho1 / Acaho2 / Mca |
| **Site name** | | | VD2 |
|  | | | |
| **Station parameters** | | | |
| **Location** | Grande Côte de Baumont | 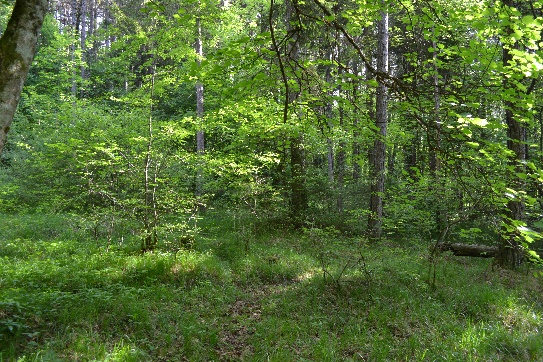 | |
| **Year of sampling** | 2016 and 2018 |  |  |
| **Altitude** | 669 m |  |  |
| **Slope** | 7° |  |  |
| **Aspect** | 110° |  |  |
| **Topography** | Slight slope |  |  |
| **Geology** | Molasse |  |  |
| **Vegetation type** | *Molinio-Pinion* |  |  |
|  | | | |
| **Soil profile** | | | |
| 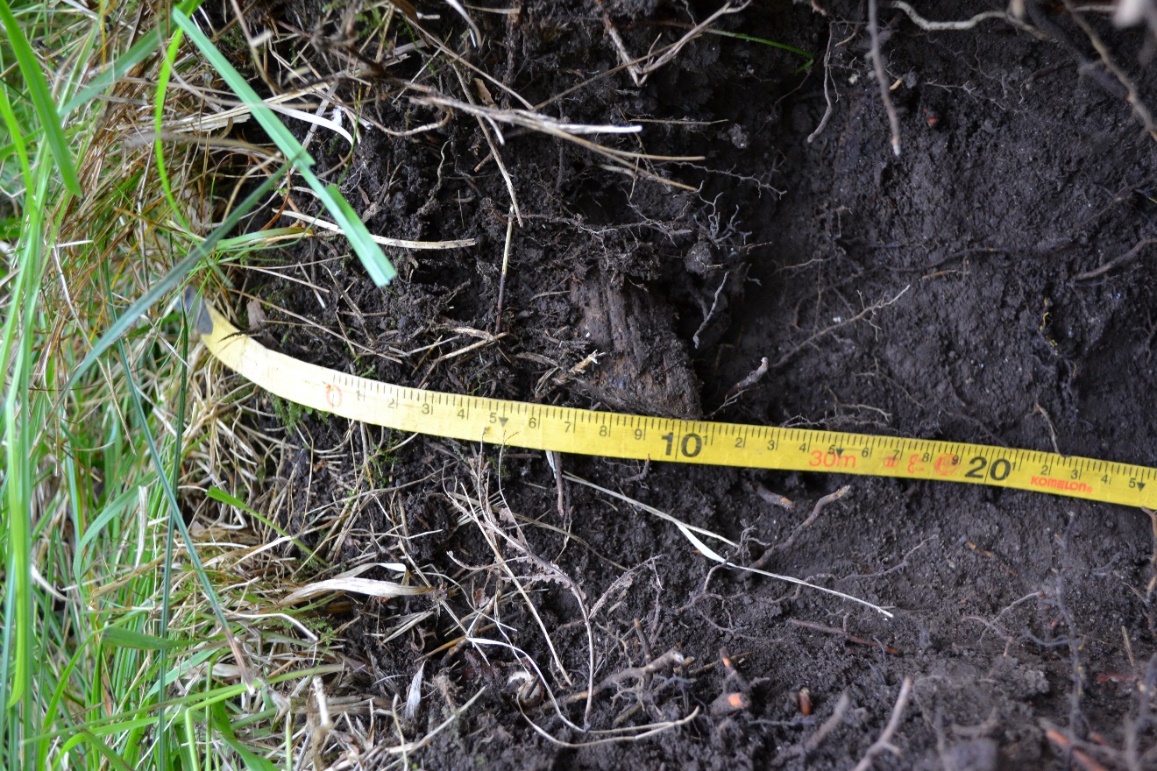 | | | |

| **Soil classification** | | | |
| --- | --- | --- | --- |
| **Soil name (Référentiel pédologique, 2009)** | | | CALCOSOL issu de Molasse |
| **Soil name (WRB, 2015)** | | | Calcaric Cambisol |
| **Humus form (Zanella et al., 2018)** | | | Mesomull |
| **Horizon sequence (Référentiel pédologique, 2009)** | | | OL / Aca-Sca / Sca-Aca |
| **Site name** | | | VD3 |
|  | | | |
| **Station parameters** | | | |
| **Location** | Vallon de la Veveyse | 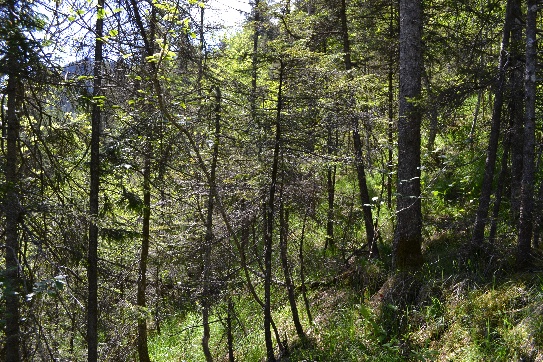 | |
| **Year of sampling** | 2018 |  |  |
| **Altitude** | 994 m |  |  |
| **Slope** | 50° |  |  |
| **Aspect** | 90° |  |  |
| **Topography** | Slope up a river |  |  |
| **Geology** | Molasse |  |  |
| **Vegetation type** | *Abieti-Fagenion* |  |  |
|  | | | |
| **Soil profile** | | | |
| 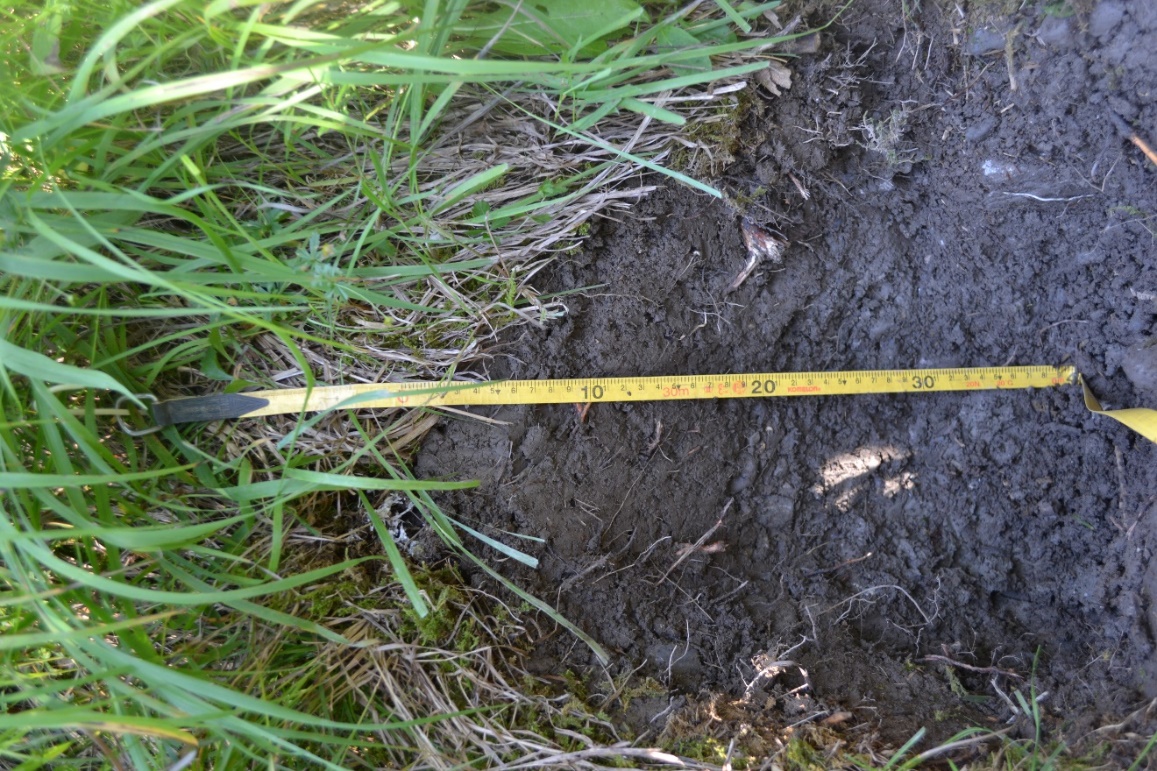 | | | |

| **Soil classification** | | | |
| --- | --- | --- | --- |
| **Soil name (Référentiel pédologique, 2009)** | | | CALCOSOL décarbonaté en surface |
| **Soil name (WRB, 2015)** | | | Calcaric Cambisol |
| **Humus form (Zanella et al., 2018)** | | | Oligomull |
| **Horizon sequence (Référentiel pédologique, 2009)** | | | OL / OF-Aci / Aci / Aca-Cca / Sca |
| **Site name** | | | VD4 |
|  | | | |
| **Station parameters** | | | |
| **Location** | Saint-George | 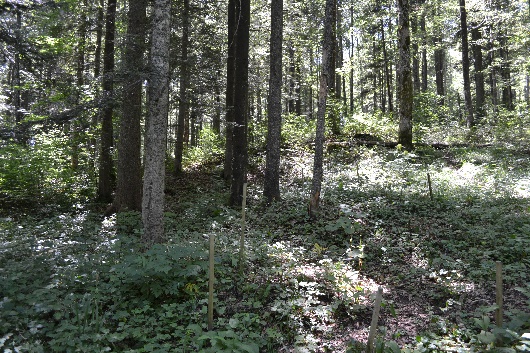 | |
| **Year of sampling** | 2016 and 2018 |  |  |
| **Altitude** | 1122 m |  |  |
| **Slope** | 15° |  |  |
| **Aspect** | 15° |  |  |
| **Topography** | Slope |  |  |
| **Geology** | NA |  |  |
| **Vegetation type** | *Abieti-Fagenion* |  |  |
|  | | | |
| **Soil profile** | | | |
| 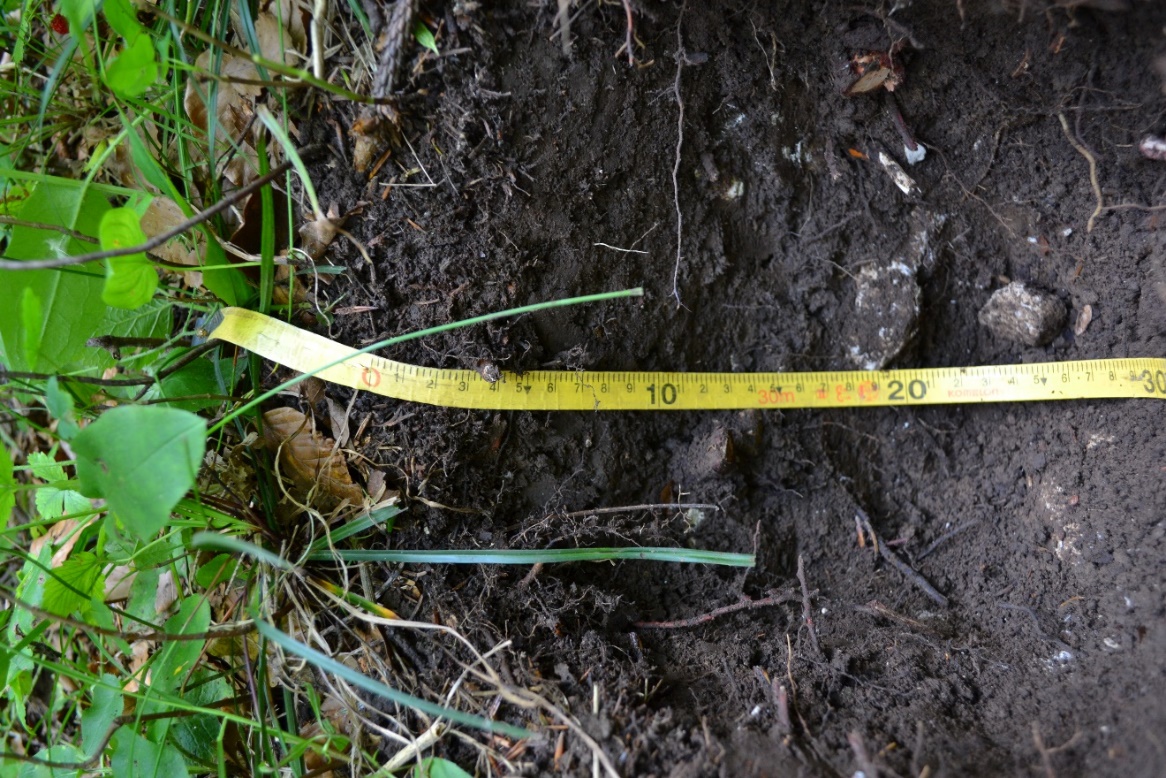 | | | |

| **Soil classification** | | | |
| --- | --- | --- | --- |
| **Soil name (Référentiel pédologique, 2009)** | | | RENDOSOL pachique issu de molasse |
| **Soil name (WRB, 2015)** | | | Rendzic Calcaric Leptosol |
| **Humus form (Zanella et al., 2018)** | | | Hemimoder |
| **Horizon sequence (Référentiel pédologique, 2009)** | | | BryoOL-vOL / OL-[BryoOL] / OF-[OH]-[vOL]-[BryoOL] / Aca |
| **Site name** | | | VD5 |
|  | | | |
| **Station parameters** | | | |
| **Location** | Fey | 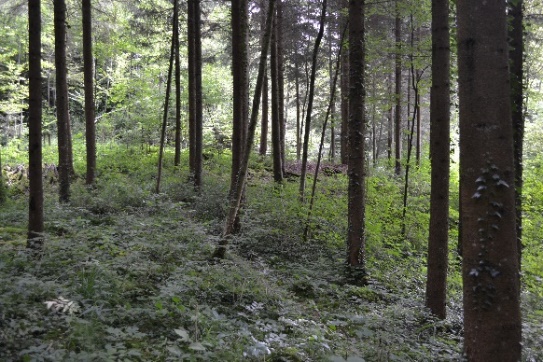 | |
| **Year of sampling** | 2016 and 2021 |  |  |
| **Altitude** | 621 m |  |  |
| **Slope** | 5° |  |  |
| **Aspect** | 136° |  |  |
| **Topography** | Slight slope below a molasse cliff |  |  |
| **Geology** | Molasse |  |  |
| **Vegetation type** | *Galio-Fagenion* |  |  |
|  | | | |
| **Soil profile** | | | |
| 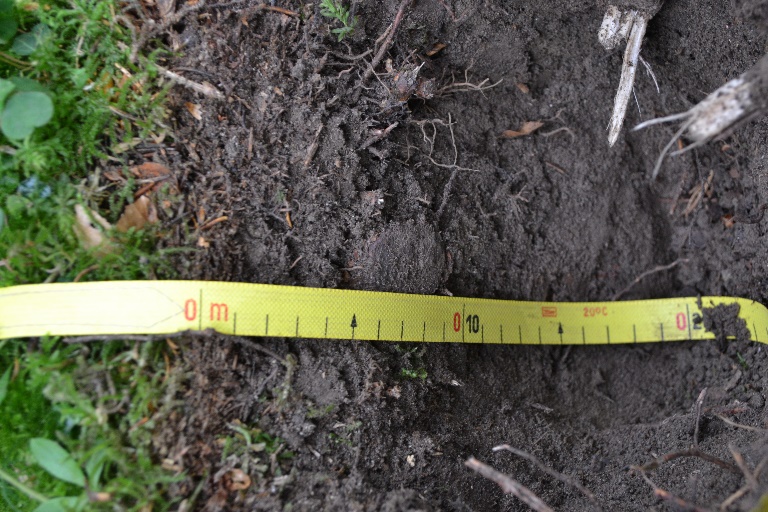 | | | |

| **Soil classification** | | | |
| --- | --- | --- | --- |
| **Soil name (Référentiel pédologique, 2009)** | | | CALCOSOL leptique |
| **Soil name (WRB, 2015)** | | | Leptic Calcaric Cambisol |
| **Humus form (Zanella et al., 2018)** | | | Dysmull |
| **Horizon sequence (Référentiel pédologique, 2009)** | | | nOL-[vOL] / OF / [OF]-Aca / Sca1 / Sca2 |
| **Site name** | | | VS1 |
|  | | | |
| **Station parameters** | | | |
| **Location** | Sion | 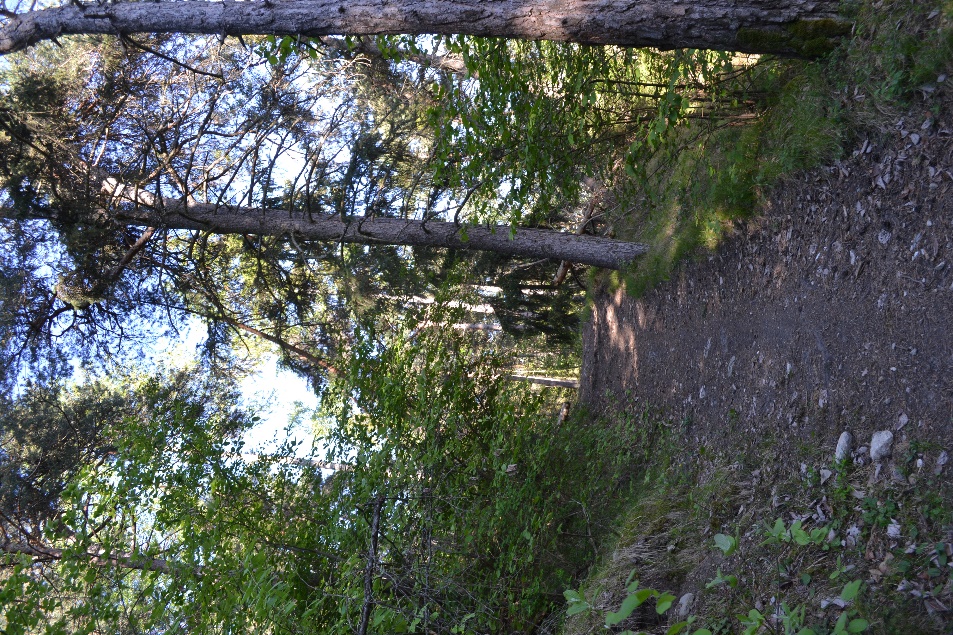 | |
| **Year of sampling** | 2019 |  |  |
| **Altitude** | 731 m |  |  |
| **Slope** | 28° |  |  |
| **Aspect** | 14° |  |  |
| **Topography** | Edge of a forest path |  |  |
| **Geology** | NA |  |  |
| **Vegetation type** | *Cephalanthero-Fagenion* |  |  |
|  | | | |
| **Soil profile** | | | |
| 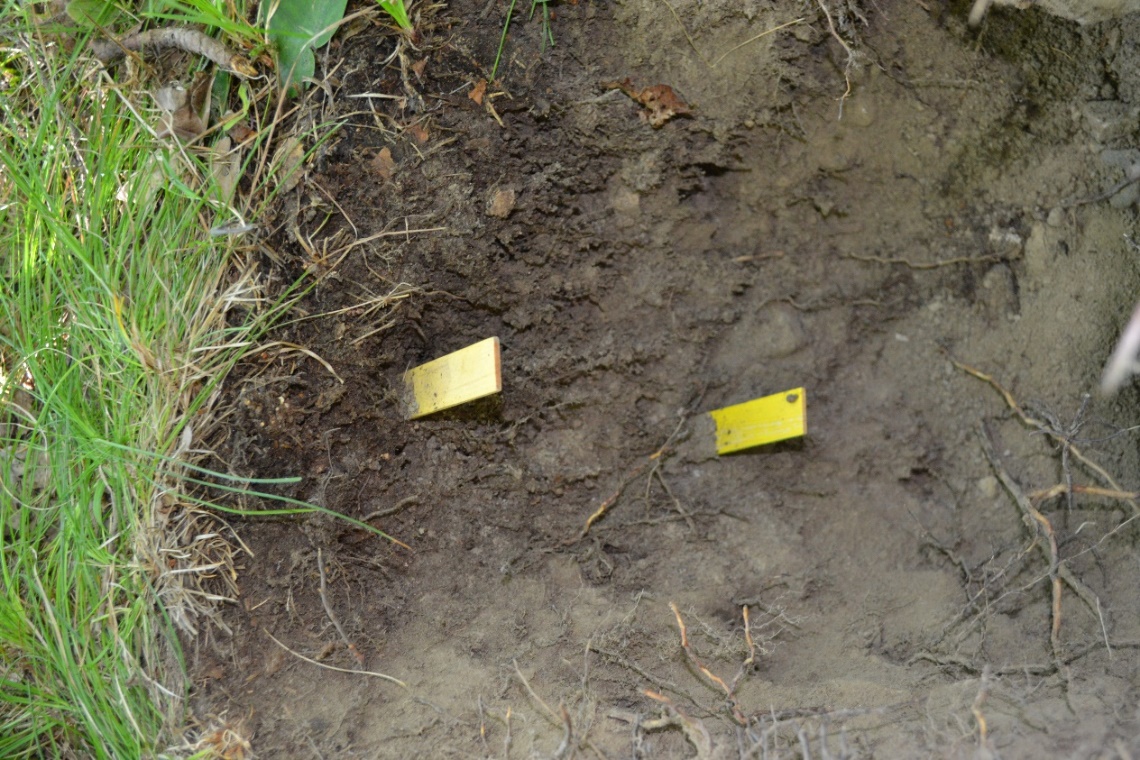 | | | |

| **Soil classification** | | | |
| --- | --- | --- | --- |
| **Soil name (Référentiel pédologique, 2009)** | | | CALCOSOL rédoxique |
| **Soil name (WRB, 2015)** | | | Stagnic Calcaric Cambisol |
| **Humus form (Zanella et al., 2018)** | | | Dysmoder |
| **Horizon sequence (Référentiel pédologique, 2009)** | | | vOL / OF-OH / Agca / Agca-Sgca |
| **Site name** | | | VS2 |
|  | | | |
| **Station parameters** | | | |
| **Location** | L’Ermitage | 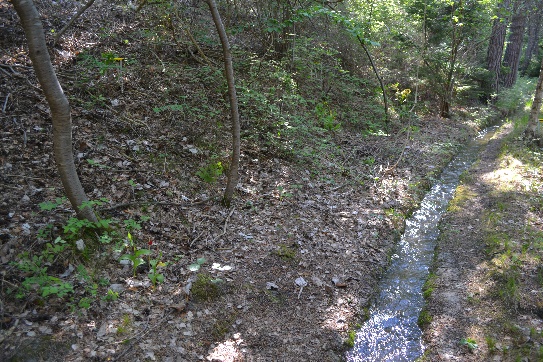 | |
| **Year of sampling** | 2019 |  |  |
| **Altitude** | 571 m |  |  |
| **Slope** | 17° |  |  |
| **Aspect** | 217° |  |  |
| **Topography** | Next to a water chanel (bisse) |  |  |
| **Geology** | NA |  |  |
| **Vegetation type** | *Cephalanthero-Fagenion* |  |  |
|  | | | |
| **Soil profile** | | | |
| 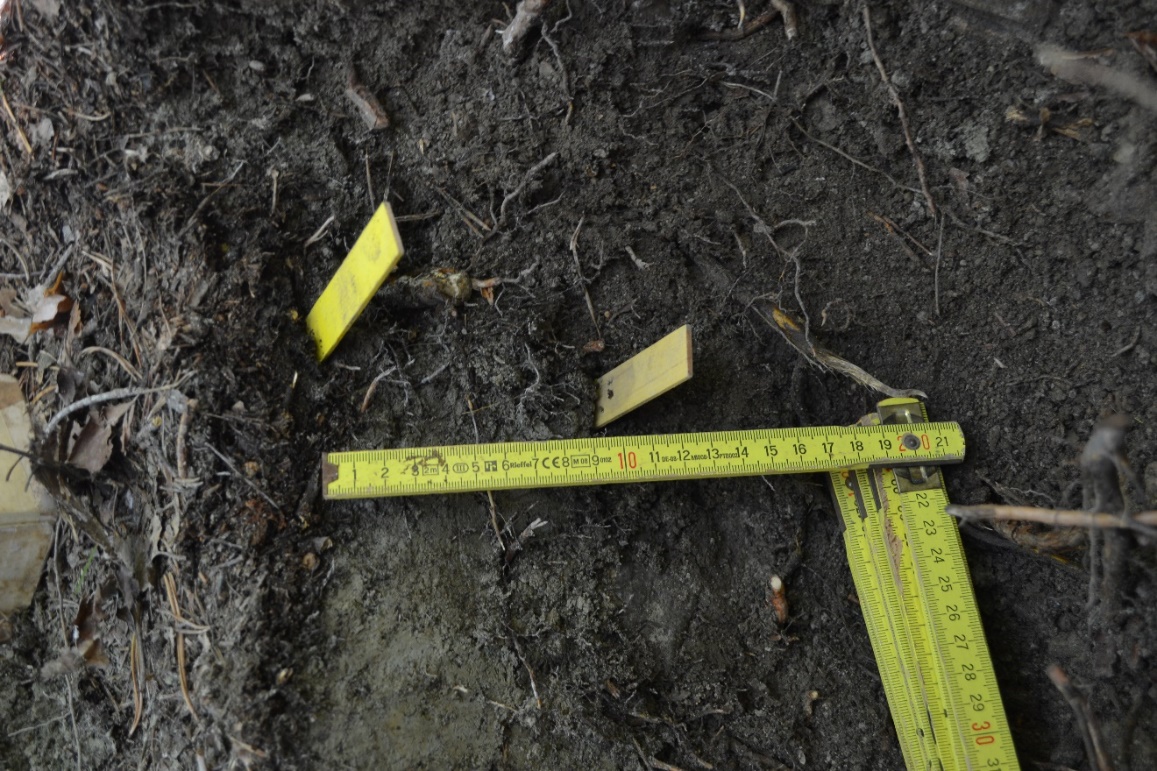 | | | |

| **Soil classification** | | | |
| --- | --- | --- | --- |
| **Soil name (Référentiel pédologique, 2009)** | | | CALCOSOL |
| **Soil name (WRB, 2015)** | | | Calcaric Cambisol |
| **Humus form (Zanella et al., 2018)** | | | Hemimoder |
| **Horizon sequence (Référentiel pédologique, 2009)** | | | [nOL]-vOL / S*-OF-[OH] / Aca-Sca / Dca |
| **Site name** | | | VS3 |
|  | | | |
| **Station parameters** | | | |
| **Location** | Forêt de Sierre | 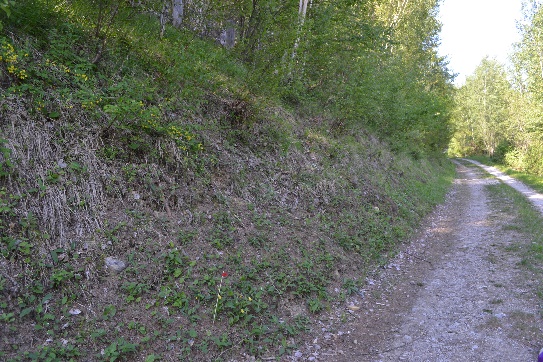 | |
| **Year of sampling** | 2019 |  |  |
| **Altitude** | 1075 m |  |  |
| **Slope** | 31° |  |  |
| **Aspect** | 354° |  |  |
| **Topography** | Edge of a forest road |  |  |
| **Geology** | NA |  |  |
| **Vegetation type** | *Abieti-Piceion* |  |  |
|  | | | |
| **Soil profile** | | | |
| 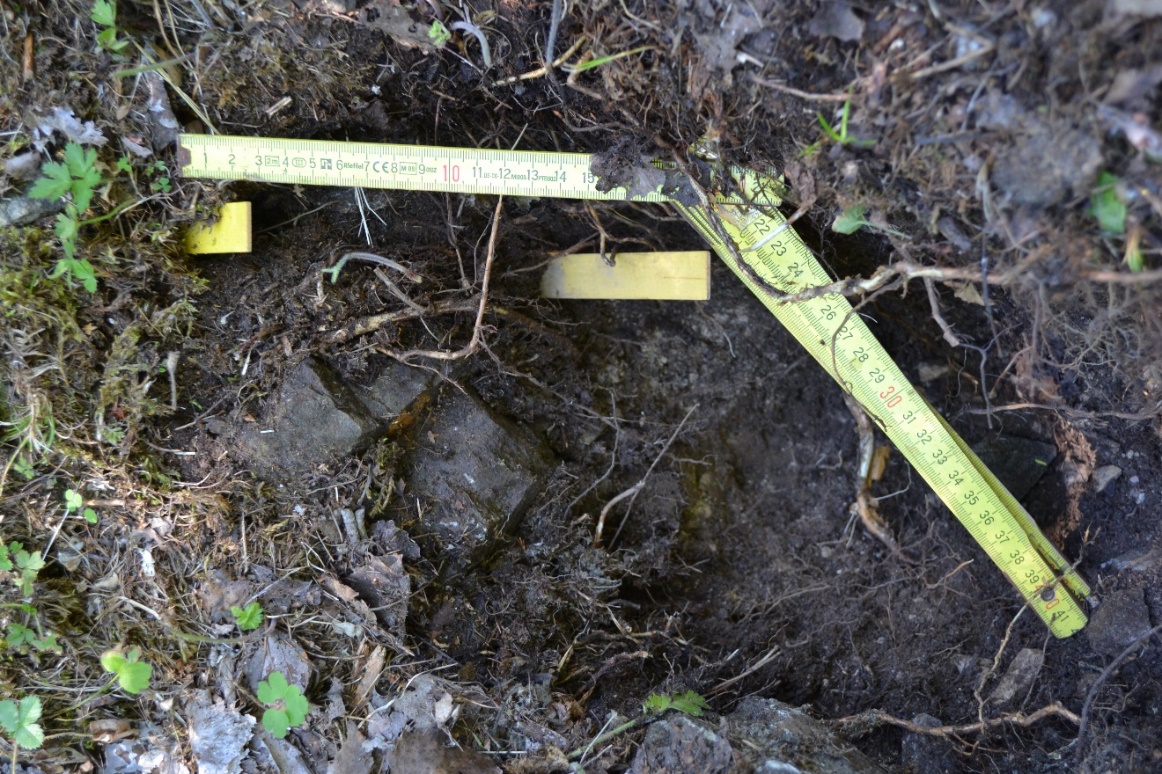 | | | |

| **Soil classification** | | | |
| --- | --- | --- | --- |
| **Soil name (Référentiel pédologique, 2009)** | | | RENDOSOL colluvial, issu d’éboulis calcaires |
| **Soil name (WRB, 2015)** | | | Calcaric Leptosol (Colluvic) |
| **Humus form (Zanella et al., 2018)** | | | Dysmull |
| **Horizon sequence (Référentiel pédologique, 2009)** | | | [nOL]-vOL / OF / Aca1 / Aca2 / Dca |
| **Site name** | | | VS4 |
|  | | | |
| **Station parameters** | | | |
| **Location** | Niouc | 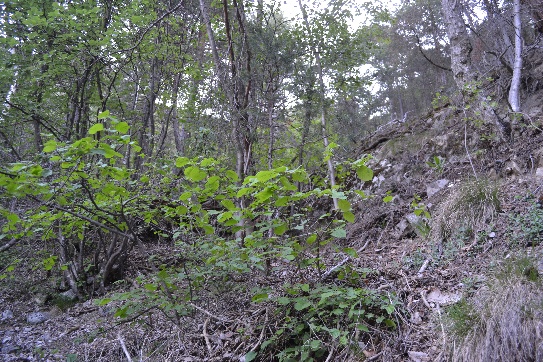 | |
| **Year of sampling** | 2019 |  |  |
| **Altitude** | 1040 m |  |  |
| **Slope** | 30° |  |  |
| **Aspect** | 278° |  |  |
| **Topography** | Bottom of a steep slope |  |  |
| **Geology** | Calcareous scree |  |  |
| **Vegetation type** | *Cephalanthero-Fagenion* |  |  |
|  | | | |
| **Soil profile** | | | |
| 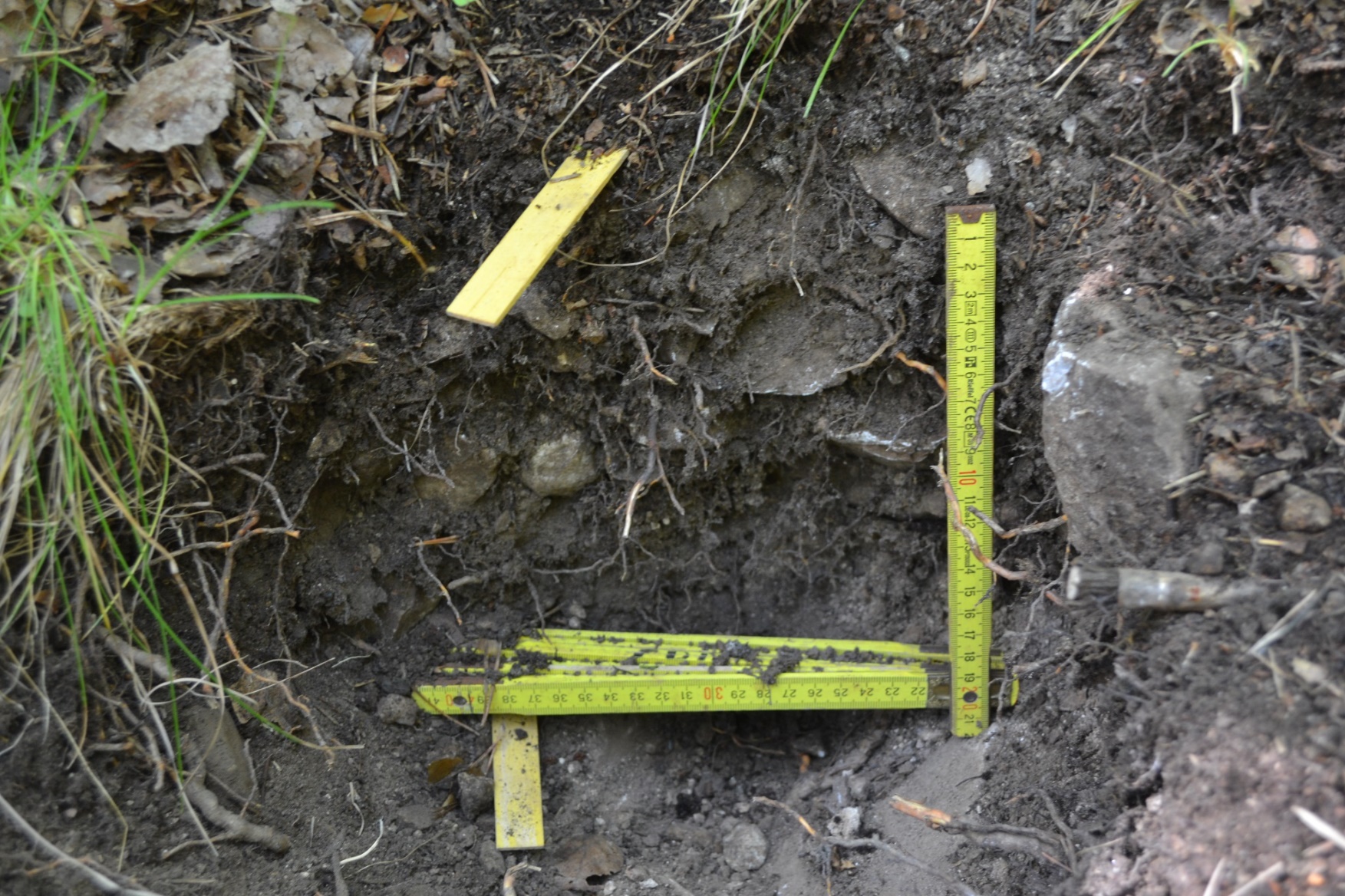 | | | |

| **Soil classification** | | | |
| --- | --- | --- | --- |
| **Soil name (Référentiel pédologique, 2009)** | | | CALCOSOL |
| **Soil name (WRB, 2015)** | | | Calcaric Cambisol |
| **Humus form (Zanella et al., 2018)** | | | Oligomull |
| **Horizon sequence (Référentiel pédologique, 2009)** | | | nOL-[vOL] / S*-OF / Aca / Sca |
| **Site name** | | | VS5 |
|  | | | |
| **Station parameters** | | | |
| **Location** | Les Diablerets | 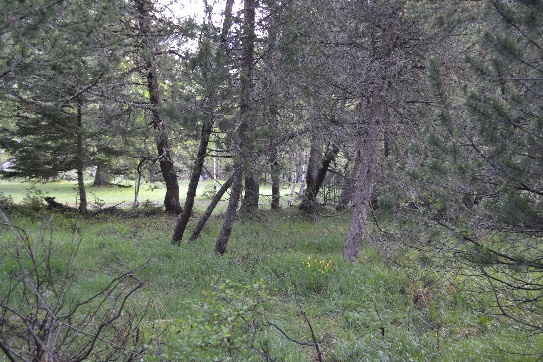 | |
| **Year of sampling** | 2019 |  |  |
| **Altitude** | 1450 m |  |  |
| **Slope** | 6° |  |  |
| **Aspect** | 190° |  |  |
| **Topography** | Flat area |  |  |
| **Geology** | NA |  |  |
| **Vegetation type** | *Erico-Pinion unicinatae* |  |  |
|  | | | |
| **Soil profile** | | | |
| 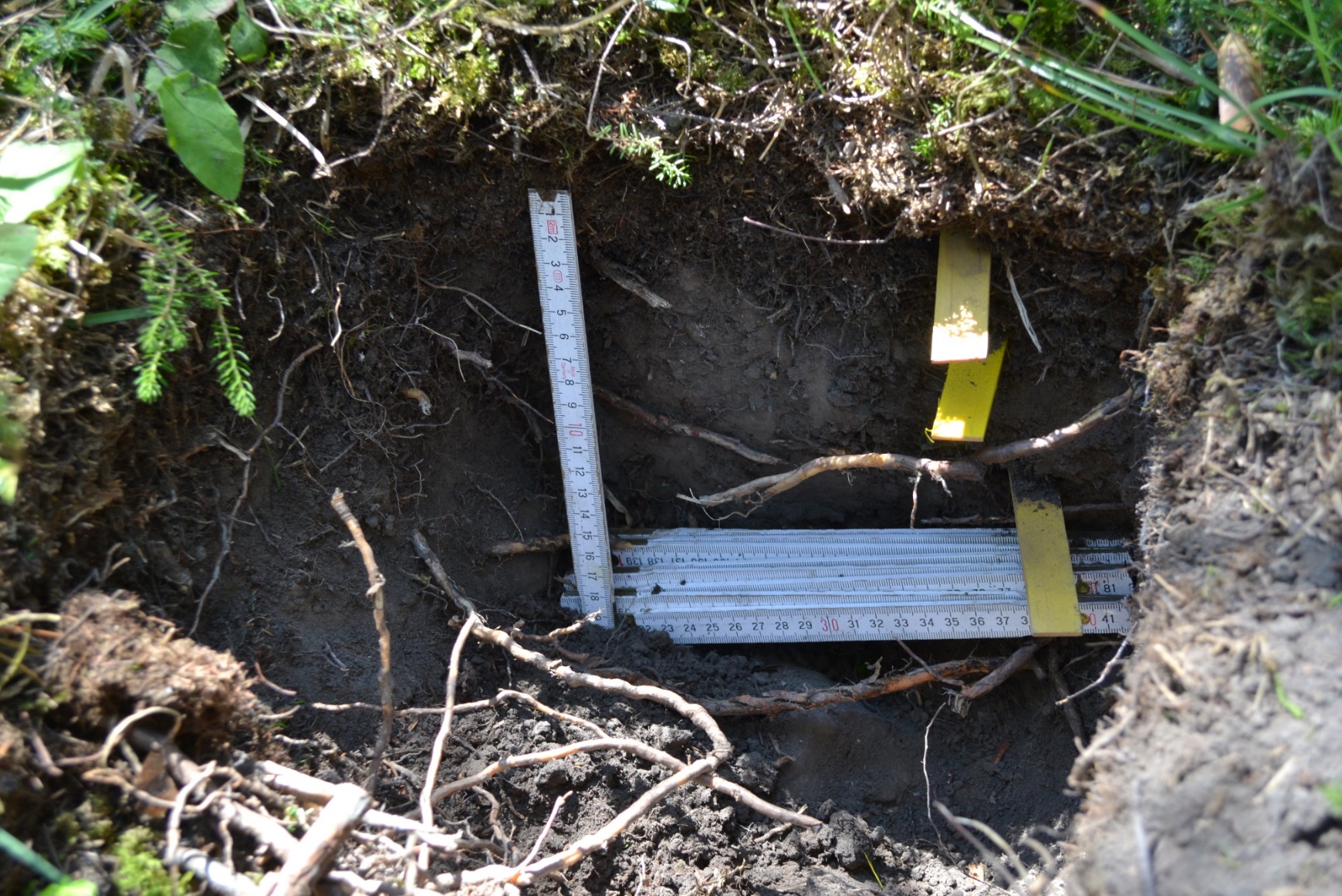 | | | |

**References**

Baize D, Girard M-C (2009) Référentiel pédologique 2008. Editions Quae, Versaille , France.

Delarze R, Gonseth Y, Eggenberg S, Vust M (2015) Guide des milieux naturels de Suisse: écologie, menaces, espèces caractéristiques. Rossolis, Bussigny, Switzerland.

IUSS Working Group W (2015) World reference base for soil resources 2014, update 2015. World Soil Resources Report 106.

Zanella A, Ponge J-F, Jabiol B, Sartori G, Kolb E, Le Bayon R-C, Gobat J-M, Aubert M, De Waal R, Van Delft B (2018) Humusica 1, article 5: Terrestrial humus systems and forms—Keys of classification of humus systems and forms. Applied Soil Ecology 122: 75-86.
